# Combinatorial nanococktails via self-assembling lipid prodrugs for synergistically overcoming drug resistance and effective cancer therapy

**DOI:** 10.1186/s40824-022-00249-7

**Published:** 2022-01-31

**Authors:** Tongyu Li, Weiwei Shi, Jie Yao, Jingyun Hu, Qiong Sun, Jing Meng, Jian Wan, Haihan Song, Hangxiang Wang

**Affiliations:** 1 The First Affiliated Hospital, Zhejiang University School of Medicine; NHC Key Laboratory of Combined Multi-Organ Transplantation; Key Laboratory of Organ Transplantation, Research Center for Diagnosis and Treatment of Hepatobiliary Diseases, Zhejiang Province Hangzhou, People’s Republic of China; 2 Department of Oncology, The First Medical Center, Chinese PLA General Hospital, Beijing, People’s Republic of China; 3 Department of Chemical Engineering, Zhejiang University, Zhejiang Province Hangzhou, People’s Republic of China; 4 Central Lab, Shanghai Key Laboratory of Pathogenic Fungi Medical Testing, Shanghai Pudong New Area People’s Hospital, Shanghai, People’s Republic of China; 5 Department of Emergency and Critical Care Medicine, Shanghai Pudong New Area People’s Hospital, Shanghai, People’s Republic of China

**Keywords:** Lipid prodrug, Self-assembly, Nanococktail, Chemoresistance, Synergism

## Abstract

**Background:**

Combinatorial systemic chemotherapy is a powerful treatment paradigm against cancer, but it is fraught with problems due to the emergence of chemoresistance and additive systemic toxicity. In addition, coadministration of individual drugs suffers from uncontrollable pharmacokinetics and biodistribution, resulting in suboptimal combination synergy.

**Methods:**

Toward the goal of addressing these unmet medical issues, we describe a unique strategy to integrate multiple structurally disparate drugs into a self-assembling nanococktail platform. Conjugation of a polyunsaturated fatty acid (e.g., linoleic acid) with two chemotherapies generated prodrug entities that were miscible with tunable drug ratios for aqueous self-assembly. In vitro and in vivo assays were performed to investigate the mechanism of combinatorial nanococktails in mitigating chemoresistance and the efficacy of nanotherapy.

**Results:**

The coassembled nanoparticle cocktails were feasibly fabricated and further refined with an amphiphilic matrix to form a systemically injectable and PEGylated nanomedicine with minimal excipients. The drug ratio incorporated into the nanococktails was optimized and carefully examined in lung cancer cells to maximize therapeutic synergy. Mechanistically, subjugated resistance by nanococktail therapy was achieved through the altered cellular uptake pathway and compromised DNA repair via the ATM/Chk2/p53 cascade. In mice harboring cisplatin-resistant lung tumor xenografts, administration of the nanococktail outperformed free drug combinations in terms of antitumor efficacy and drug tolerability.

**Conclusion:**

Overall, our study provides a facile and cost-effective approach for the generation of cytotoxic nanoparticles to synergistically treat chemoresistant cancers.

**Supplementary Information:**

The online version contains supplementary material available at 10.1186/s40824-022-00249-7.

## Introduction

Systemic chemotherapy remains the mainstay therapeutic modality for advanced and metastatic cancers and has shown efficacy in extending patient survival. However, the emergence of de novo or acquired chemoresistance not only discounts the clinical response but also represents one of the formidable obstacles for effective cancer chemotherapy [1, 2]. To tackle these challenges, combinatorial regimens that integrate multimodal therapies have been extensively explored and have been remarkably successful in the clinic. Unlike single-agent therapies, combinatorial strategies harness multiple synergistic therapeutics to rewire distinct signaling pathways in cancer [3, 4]. Unfortunately, the clinical use of combinatorial regimens remains a significant challenge because coadministration of individual free drugs suffers from different pharmacokinetics and dissimilar biodistributions, as well as additive toxicity risks [5–7]. Therapeutic delivery with nanoparticle approaches holds the potential to offer effective treatments against a wide range of indications, from cancer therapy to vaccination [8–10]. Currently, some nanotherapeutics have been clinically approved, and more are tested in clinical trials at different stages [11–13]. In particular, coencapsulation within a single nanoparticle platform has been extensively explored in numerous preclinical models [14–16]. These codelivery strategies not only help minimize off-target dissemination of toxic agents but also maximize the treatment synergism by codelivery to the same target cells [17]. However, translation of such a nanosystem to clinically successful medicines remains a considerable challenge due to some intrinsic flaws, including complicated fabrication processes, high variability in in vivo performance, and essential requirements of exogenous excipients [18, 19].

Supramolecular chemistry leveraging noncovalent interactions between individual molecular building blocks provides new opportunities for the construction of therapeutically available nanoassemblies [20]. As a pioneering example, the use of a squalene moiety to chemically derive anticancer chemotherapies has been developed, which results in a new generation of small molecular nanotherapies [21, 22]. We recently also showed that the attachment of drug molecules to natural omega-3 polyunsaturated fatty acids (PUFAs) through bioactivatable linkages generates new prodrug entities, which confers upon them the ability to self-assemble in aqueous media without the need for exogenous excipients [23, 24]. This approach renders prodrug nanoassemblies with substantially improved in vivo antitumor efficacy and reduced toxicity relative to their free drug forms. Exploiting “PUFAylation” technology, we have successfully transformed several therapeutics, including immunosuppressive agents, into self-deliverable nanotherapies against cancer and acute graft rejection [25–27]. In addition, structurally dissimilar drugs are expected to be tolerable in this approach for forming codelivery systems. To date, no combinatorial nanotherapies based on therapeutic PUFAylation have been explored. Here, we hypothesized that PUFAylated drug coassembled nanosystems may be able to mitigate chemoresistance via rationally optimized synergism, improve in vivo performance by synchronized pharmacokinetics, and reduce undesired toxicity.

Platinum-based chemotherapeutics are widely used in the clinic for the treatment of advanced cancer [28, 29]. Unfortunately, only a subset of cancer patients respond to platinum agents but still experience the emergence of drug resistance and substantial systemic toxicity, which deters their clinical application [30, 31]. Consequently, a second agent that increases the efficacy of platinum agents but does not have additive toxicity may have great benefits for improving treatment outcomes. Published reports suggest that combining irinotecan (CPT-11) with cisplatin had the desired response rate (45% on average) in patients with advanced non-small cell lung cancer (NSCLC) [32]. Such a combination regimen takes advantage of different mode-of-action drugs to induce high levels of DNA lesions.

In this study, we report the development of a therapeutic nanococktail (NC) platform constructed from self-assembling small molecular lipid prodrugs and test it against cisplatin-resistant NSCLC. The NC comprises two PUFAylated chemotherapeutics that are constructed from cisplatin and 7-ethyl-10-hydroxy-camptothecin (SN38) via conjugation with linoleic acid (LA). The self-assembled NC can be further refined with 1,2-distearoyl-*sn*-glycero-3-phosphoethanolamine-*N*-[methoxy-(polyethyleneglycol)2000] (DSPE-PEG_2k_), an amphiphilic matrix, to form a systemically injectable and PEGylated nanoparticle with minimal excipients. PEGylated NC exhibits spherical nanostructures with sub-60 nm diameters and is stable in aqueous solutions. In cells, NCs can release active platinum (II) and SN38 agents in response to intracellular reducing agents (e.g., glutathione, GSH) and esterase, respectively [30, 33].

The drug ratios incorporated into the NC for maximum synergy were optimized by calculating the combination index (CI), and the synergistic effect of the NC was carefully examined in cisplatin-resistant A549^cisR^ cells. Transporter-independent internalization of the NC via endocytosis contributed to enhanced cytotoxicity compared with free drugs. In vitro mechanistic studies demonstrated that the remarkable synergism was attributable to the negative modulatory role of the SN38 agent through the inhibition of the DNA repair mechanism and ATM/Chk2/p53-mediated pathway. Animal experiments further revealed that NC outperformed the free drug combination in terms of antitumor efficacy and drug tolerability. Overall, the study highlights that coassembling cisplatin and SN38 derivatives via a PUFAylation approach possessed superiority for safe in vivo delivery and was potent enough to eliminate resistant A549^cisR^ cells.

## Results

### Synthesis of PUFAylated prodrugs and characterization of nanoassemblies

**Fig. 1:**
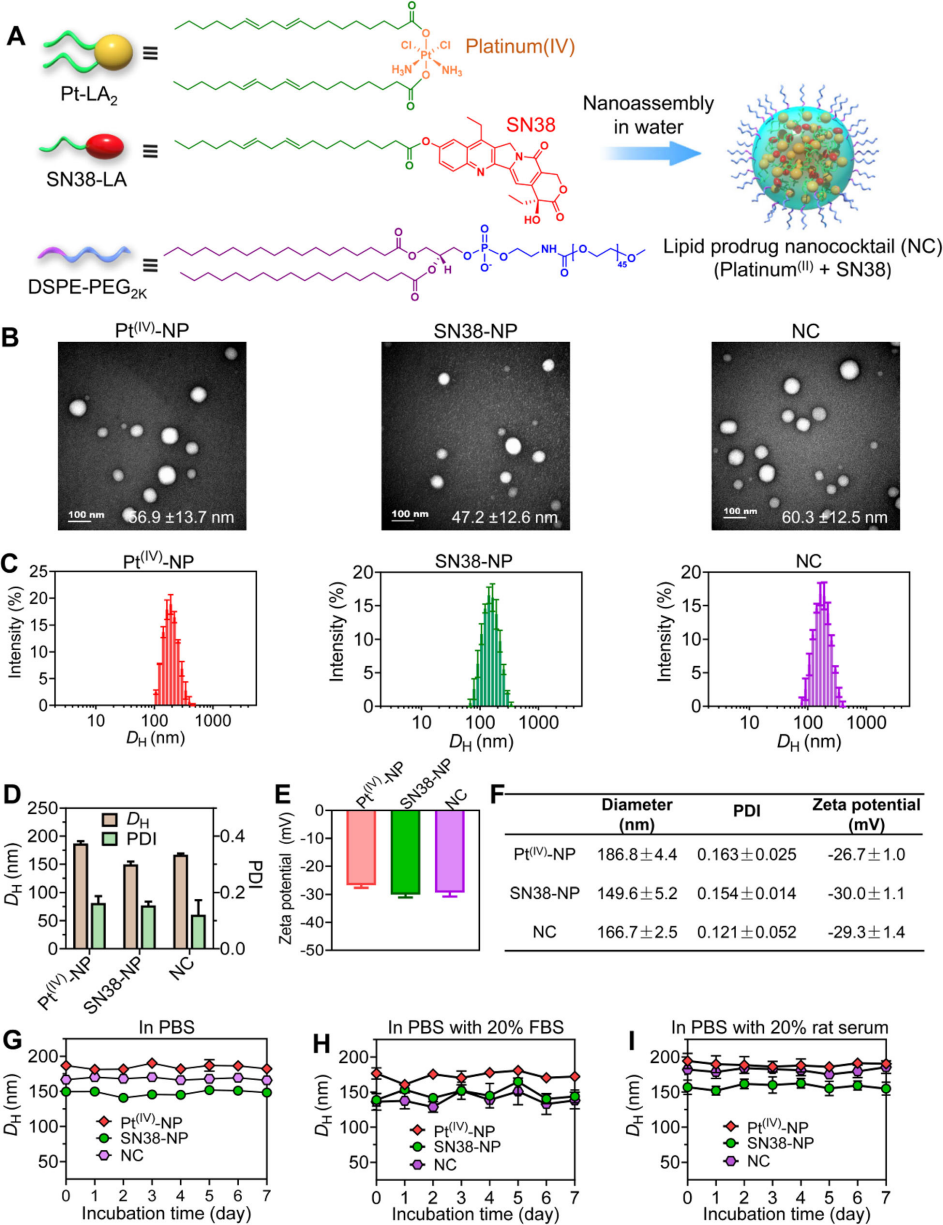
Characterization of PEGylated prodrug-loaded nanoparticles. (**A**) The nanococktail from coassembling platinum (IV) and SN38 derivatives was prepared in an aqueous solution for intravenous injection. (**B**) TEM images of Pt^(IV)^-NP, SN38-NP, and the nanoparticle cocktail (the molar ratio of Pt-LA_2_ to SN38-LA prodrugs was fixed at 5:1). Scale bars, 100 nm. (**C**-**E**) Distribution of nanoparticle sizes (**C**), average hydrodynamic diameters and PDI (**D**), and zeta potentials (**E**), determined by DLS measurement. (**F**) Characterization of prodrug-formulated nanoparticles. (**G**-**I**) Assessment of nanoparticle stability in PBS (pH = 7.4) (**G**), in PBS supplemented with 20% fetal bovine serum (FBS) (**H**), or in PBS supplemented with 20% rat serum (**I**) over one week

LA was strategically used as a pro-moiety to couple with oxidated platinum (IV) and SN38 via esterification to generate Pt-LA_2_ and SN38-LA prodrugs, respectively (Fig. 1A and Fig. S1–10) [30, 33]. The prodrug constructs are sufficiently amphiphilic to assemble in aqueous solutions via a reprecipitation method, forming water-soluble nanoparticles. Surface cloaking of PEGylation is expected to reduce clearance by the mononuclear phagocyte system (MPS) and to prevent them from being degraded in the blood [34]. Thus, amphiphilic DSPE-PEG_2k_ lipid was utilized to cloak the particle surface to produce injectable nanoparticles. Moreover, due to the prodrug self-assembly capacity, excipients for drug formulation can be minimized. Injection of the mixture of prodrug/matrix (10:1, w/w, dissolved in dimethyl sulfoxide (DMSO)) into deionized (DI) water generated individual nanosuspensions. Further removal of DMSO via dialysis did not cause visible precipitates, indicating that the improved solubility of the nanoassemblies was independent of organic solvents. The resultant assemblies were subjected to transmission electron microscopy (TEM) observation and dynamic light scattering (DLS) analysis (Fig. 1B-D). In TEM images, both nanoassemblies had uniformly spherical structures (termed Pt^(IV)^-NP and SN38-NP, Fig. 1B). DLS analysis confirmed that these nanoassemblies showed monomodal distributions with relatively low polydispersity indices (PDI < 0.2) (Fig. 1C).

We further verified whether the Pt-LA_2_ and SN38-LA prodrugs can be coassembled into a single nanoparticle cocktail with the aid of the DSPE-PEG_2k_ matrix (Fig. 1A and B). Both compounds were completely miscible, and the coassembled NC was shaped in spherical nanostructures, similar to the nanoassemblies prepared from individual prodrugs (Fig. 1B), which was also evidenced by the DLS results (Fig. 1C). All constructed nanoparticles had negative ζ potentials, indicating that they are refractory to adsorption to blood components with negative charges (Fig. 1E and F). In addition, stability testing suggested that PEGylated nanoassemblies neither varied in diameter nor precipitated during one week of observation in phosphate-buffered saline (PBS) or in PBS containing serum at 37 °C (Fig. 1G, H, and I). Together, these results all indicated that the combinatorial nanoassemblies were successfully constructed, with tunable drug ratios for maximizing the synergism in the scaffold. Increased water solubility renders the NC applicable for preclinical studies via intravenous injection.

### Synergistic activity of cocktail nanoassemblies in cisplatin-resistant A549^cisR^ cells

Cellular uptake of cisplatin strongly depends on copper transport protein 1 (Ctr1) located on the membrane, and downregulation of Ctr1 is one of the major contributors associated with platinum drug resistance [35–37]. In platinum-sensitive A549 cells, Ctr1 was normally expressed; however, the expression of the Ctr1 transporter was substantially abolished in A549^cisR^ cells (Fig. S11A), which potentially makes A549^cisR^ cells resistant to cisplatin treatment. For example, A549^cisR^ cells showed ~ 5-fold higher resistance to cisplatin than A549 cells, as evidenced by the cell viability assay (Fig. S11B). Notably, self-assembled platinum (II) nanoparticles significantly reduced cell viability compared with cisplatin, showing the potential to overcome drug resistance.

**Fig. 2:**
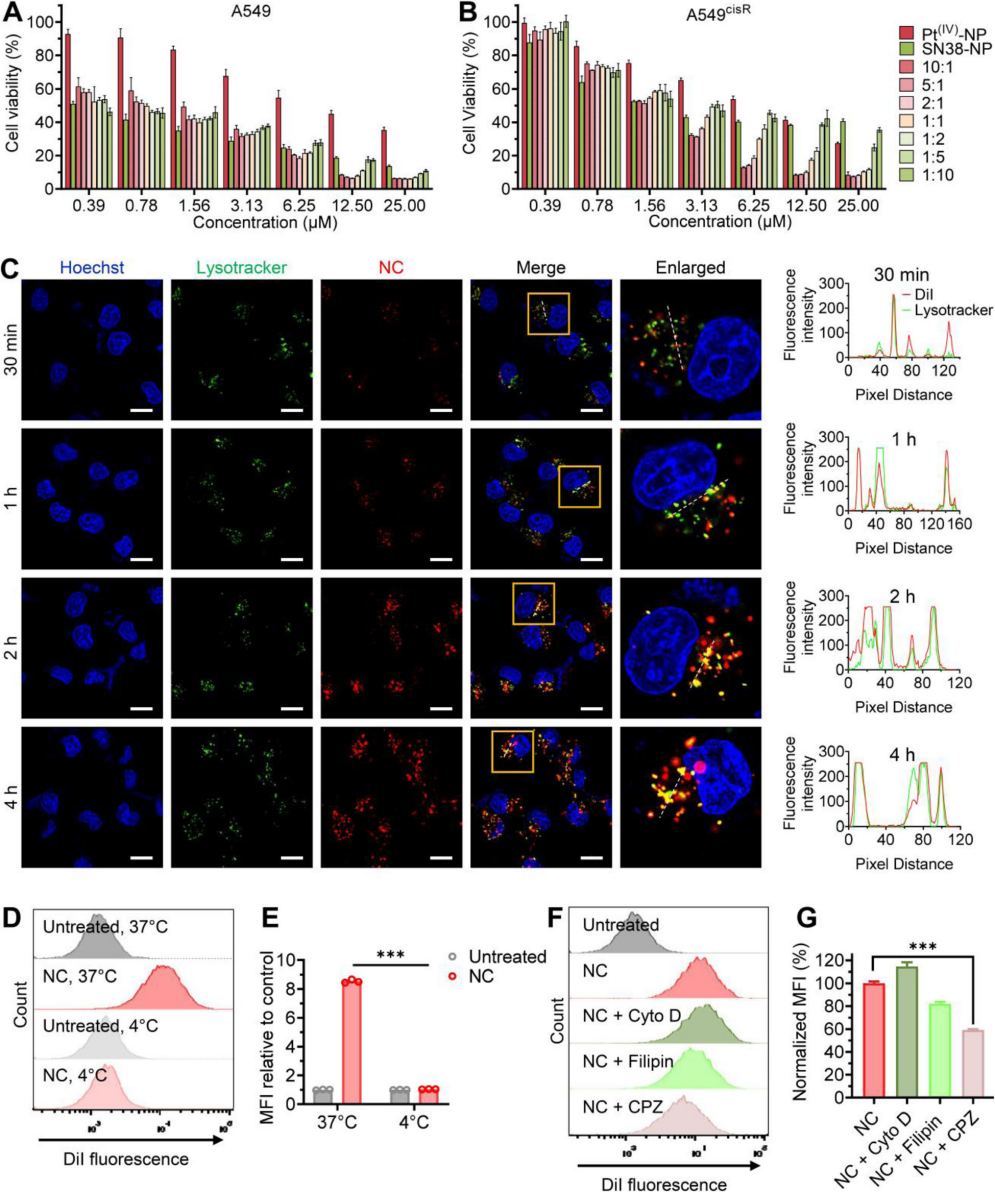
The nanococktail showed preferred synergism in resistant cancer cells. (**A** and **B**) Optimization of the nanoparticle cocktails in terms of in vitro cytotoxicity. The NCs fabricated at varying molar ratios of Pt-LA_2_ to SN38-LA prodrugs were tested for their cell-killing ability against platinum-sensitive cells and platinum-resistant cells. Drugs were added to cells for 72 h of incubation, and cell viability was measured by the CCK-8 assay. (**C**) Confocal images representing the cellular uptake of DiI-loaded combinatorial NC by A549^cisR^ cells after incubation for a predetermined duration at 37 °C. Hoechst 33342 (blue) and LysoTracker NDN-26 (green) were included to visualize the cell nuclei and lysosomes, respectively. The cellular colocalization of the NC and LysoTracker was evaluated by the distribution of their corresponding fluorescent signals using ImageJ software (right). Scale bars, 15 μm. The internalization of DiI-labeled NC into A549^cisR^ cells after incubation at either 37 °C or 4 °C for 4 h (**D**). Histogram reveals the fold change of cellular uptake at different incubation temperatures (**E**). (**F** and **G**) Fluorescence-activated cell sorting (FACS) analysis to quantitatively examine the cellular uptake of the NC. Prior to incubation with the NC, cells were treated with cytochalasin D (cyto D), filipin, or chlorpromazine (CPZ) at 37 °C for 30 min (**F**). Histogram representing the percentage of uptake as determined by FACS analysis (**G**). The data are presented as the means ± SD; ***p < 0.001

The camptothecin derivative SN38 exerts its cytotoxicity through the inhibition of nuclear DNA topoisomerase I (TOP1), thus leading to increased DNA double-strand breaks (DSBs) in a different mode of action [38]. Hence, we speculated that integrating SN38 into platinum nanoparticles could yield therapeutic synergy in resistant cells. The NCs loading with varying ratios of the Pt-LA_2_ and SN38-LA prodrugs were examined for their half-maximal inhibitory concentrations (IC_50_). In parental A549 cells, significantly low IC_50_ values were observed when increasing the ratios of SN38-NP, indicating that a higher concentration of SN38 favored platinum therapy in this cell line (Fig. 2A and Table 1). In the A549^cisR^ cells, the potency of Pt^(IV)^-NP could be augmented by the addition of SN38-NP (Fig. 2B and Table 1). Furthermore, we examined the synergism of NC delivery by calculating the combination index (CI) at half inhibition concentration among different combinations. The NC scaffolds were prepared at varying molar ratios of platinum to SN38 agents and added to cells for the cytotoxicity assay. Among these, the NCs prepared at 5:1 and 10:1 (Pt-LA_2_/SN38-LA, molar ratio) showed strong synergism in both sensitive and resistant cells (Table 1). Notably, in A549^cisR^ cells, the NC at 5:1 had the lowest CI (~ 0.271), reducing the IC_50_ to 1.67 ± 0.05 μM simultaneously. Such a high synergistic efficacy achieved at low concentrations of the NC platform would not only potentiate the therapy against resistant cancer but also alleviate side effects generally caused by chemotherapy.

**Table 1:** IC_50_ values and combination indices of Pt^(IV)^-NP, SN38-NP, and nanococktails with varying drug molar ratios against human non-small cell lung cancer cell lines

		A549		A549^cisR^	
		IC_50_ (μM)	CI (50% inhibition)	IC_50_ (μM)	CI (50% inhibition)
Pt^(IV)^-NP		9.64 ± 0.55	–	7.51 ± 0.36	–
SN38-NP		0.39 ± 0.03	–	3.91 ± 0.69	–
Nanococktail (Pt-LA_2_/SN38-LA, molar ratio)	10:1	1.15 ± 0.10	0.359	1.80 ± 0.07	0.287
	5:1	0.82 ± 0.05	0.424	1.67 ± 0.05	0.271
	2:1	0.81 ± 0.05	0.757	1.98 ± 0.07	0.400
	1:1	0.66 ± 0.07	0.952	2.51 ± 0.12	0.550
	1:2	0.65 ± 0.06	1.217	2.93 ± 0.15	0.635
	1:5	0.67 ± 0.06	1.561	4.24 ± 0.44	1.025
	1:10	0.52 ± 0.10	1.286	4.72 ± 0.81	1.316

### Cellular uptake of the nanococktail

We next investigated the cellular internalization of the NC against cancer cells. For this purpose, the fluorescent dye DiI was used to label the NC by a coassembling protocol (Fig. S12) [27]. A549^cisR^ cells were exposed to the DiI-labeled NC, and the kinetics of cellular uptake were evaluated by confocal laser-scanning microscope (CLSM) analysis. Time-lapse CLSM imaging revealed that the NCs exhibited effective cellular internalization with an increased red fluorescence signal over the time course (Fig. 2C). Colocalization studies of the NC with commercial LysoTracker Green in cells demonstrated the specific accumulation of the NC in endo/lysosomes (Fig. 2C). Upon extended incubation, a low extent of colocalization with endo/lysosomes was observed, indicating the sufficient endosomal escape capacity of the NC. To examine whether the mechanism of NC uptake was due to endocytosis, we tested uptake efficiency under different incubation conditions (i.e., temperature). Compared with effective internalization at 37 °C, lowering the temperature to 4 °C significantly reduced the uptake of the NC by A549^cisR^ cells (Fig. 2D and E), suggesting an energy-dependent endocytosis pathway.

To further elucidate the uptake mechanism, different inhibitors that specifically target endocytosis pathways were examined [39]. When A549^cisR^ cells were pretreated with chlorpromazine, an inhibitor of clathrin-mediated endocytosis, flow cytometry analysis showed that the cellular uptake of the NC was substantially decreased by ~ 40% compared to that of the untreated cells (Fig. 2F and G). Filipin also reduced the internalization of the NC to 80%, indicating that caveolae-mediated endocytosis was also involved. These results were consistent with the reported studies that in general, multiple endocytosis pathways contribute to the cellular uptake of nanoparticles [40, 41]. Distinct from cisplatin uptake, which heavily relies on the Ctr1 transporter, the NC scaffold efficiently “smuggled” the cytotoxic derivatives into the resistant cells primarily through clathrin-mediated endocytosis, which is an essential prerequisite for their intracellular synergism.

### The nanococktail blocked cell growth and induced apoptosis by triggering DSBs

**Fig. 3:**
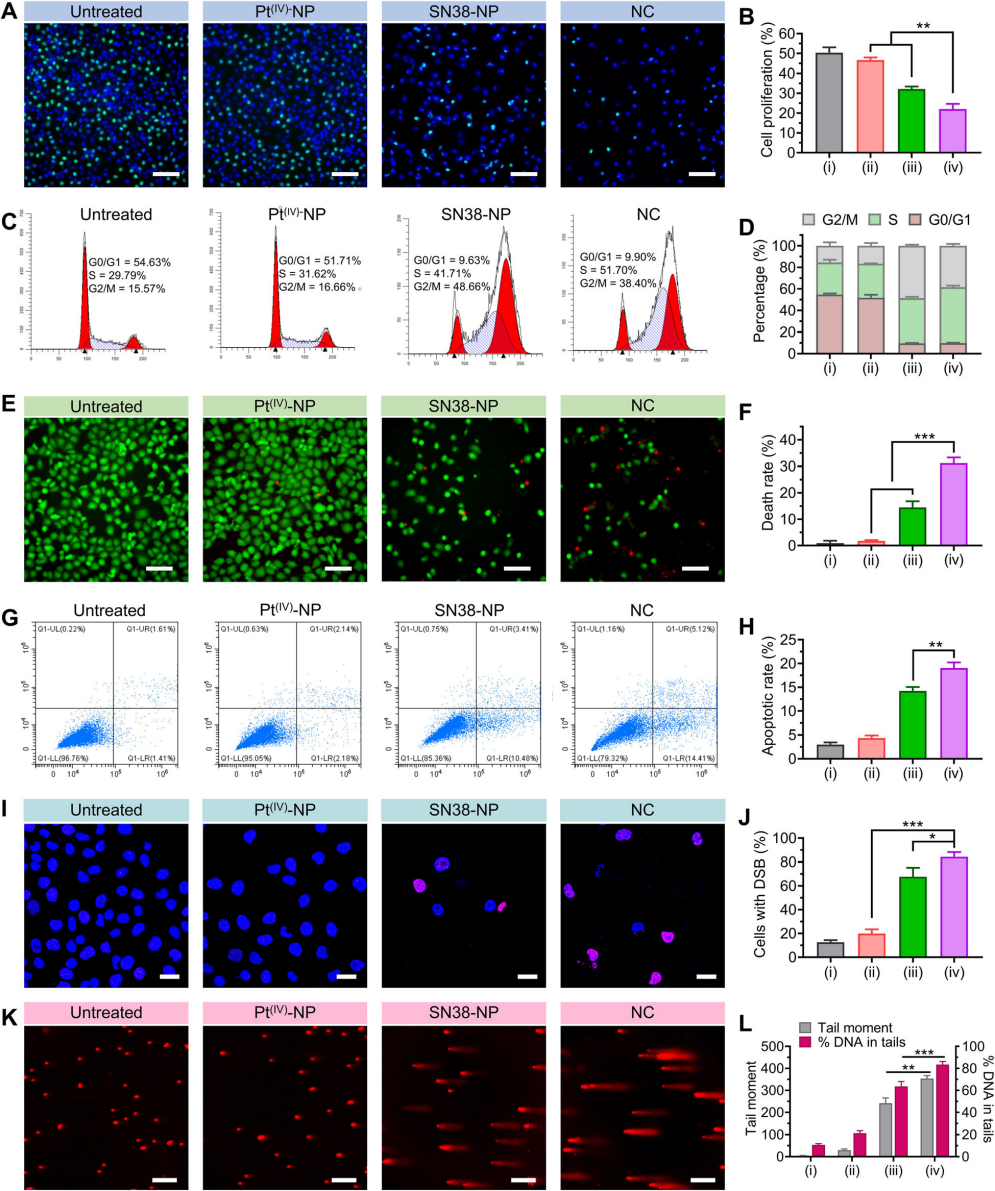
The nanococktail induced blocked cell growth and elevated apoptosis *by* triggering DSBs. (**A** and **B**) Representative images of A549^cisR^ cell proliferation after treatment with the NC fabricated with Pt-LA_2_ (5 μM) and LA-SN38 (1 μM) for 24 h. Scale bars, 100 μm. (**C** and **D**) Cell cycle distribution of A549^cisR^ cells upon drug treatments for 24 h. Cells were stained with propidium iodide (PI) and subjected to flow cytometry for DNA content analysis. The percentage of the cell population is shown in the histogram. (**E** and **F**) Fluorescence images of live/dead cells via calcein-AM/PI staining after A549^cisR^ cells were treated with the optimized NC for 24 h. Calcein-AM stained viable cells, emitting green fluorescence, while PI permeated dead cells and produced red fluorescence. Scale bars, 100 μm. (**G** and **H**) Apoptosis analysis of A549^cisR^ cells after exposure to each treatment for 24 h (**G**). The apoptotic rate was shown in the histogram (**H**). (**I** and **J**) Cells were stained with γH2AX (red) to visualize DNA damage under confocal microscopy. Scale bars, 25 μm. (**K** and **L**) Representative images showing the comet assay to quantify the degree of damaged DNA in A549^cisR^ cells. Scale bars, 200 μm. Both tail moment and %tail DNA were analyzed from 50 individual cells with Comet Assay Software Project (CASP) version 1.2.3 (**L**). The Roman numbers under the axis represent the corresponding treatments: (i) untreated, (ii) Pt^(IV)^-NP, (iii) SN38-NP, and (iv) NC. The data are presented as the means ± SD; ** *p* < 0.01, ***p < 0.001

To further validate whether the coassembled prodrug cocktail could inhibit the proliferation of A549^cisR^ cells, we used a 5-ethynyl-2′-deoxyuridine (EdU) incorporation assay to assess DNA replication. As shown in Fig. 3A and B, treatment with Pt^(IV)^-NP (5 μM, cisplatin equivalence) showed negligible antiproliferative activity in A549^cisR^ cells, presumably due to drug resistance. In contrast, SN38-NP (1 μM, SN38 equivalence) induced a significant reduction in the EdU-positive rate, and this effect was profound when the cells were exposed to NC treatment. Because decreased cell proliferation reflects delayed cell cycle progression, we examined the cell cycle profiles of A549^cisR^ cells by flow cytometry analysis (Fig. 3C). Pt^(IV)^-NP monotherapy did not alter the cell cycle. However, remarkable cells were arrested in both S and G_2_/M phases after SN38-NP treatment. Of note, we observed a high level of S phase when the cells were treated with NC (51.7% for NC versus 41.7% for SN38-NP monotherapy, Fig. 3D). These data were in accordance with the mode of action of cisplatin and SN38, both of which cause double-strand DNA breakage during DNA synthesis and arrest cells in S and G_2_ phases [38, 42]. Hence, SN38-NP appeared to produce a specific synergistic combination with Pt^(IV)^-NP to impose DNA damage against cisplatin-resistant cells. TOP1 inhibition by a camptothecin prodrug may potentially incur elevated DNA-Pt adducts in cells.

The cell death/survival profile upon NC treatment was analyzed through calcein-AM/PI dual staining. Fluorescence imaging showed that a large proportion (~ 30%) of dead A549^cisR^ cells appeared upon NC treatment, confirming that NCs fabricated at the optimized ratio of 5:1 potently induced cell death (Fig. 3E and F). A fluorescein Annexin V/propidium iodide colabeling assay was further conducted to quantify cell apoptosis. As a result, Pt^(IV)^-NP-treated cells did not show any apparent difference in apoptotic rates from untreated cells. In contrast, cells treated with SN38-NP exhibited a high fraction of apoptosis, in which apoptotic cells accounted for 14.2% of the total cells and further increased to 19.1% after NC treatment (Fig. 3G and H). These data paralleled the trends in the cell cycle distribution, suggesting that arrested cells caused by NCs further underwent increased apoptosis.

The observed synergy of the NC to provoke robust apoptosis can be explained by the nonoverlapping mechanisms of cisplatin and SN38. Phosphorylated H2A histone family member X (γH2AX) is a marker for DNA DSBs and represents an onset event in DNA damage responses [43]. We, therefore, analyzed the level of γH2AX to investigate NC-induced DNA damage. Consistent with the insufficient cytotoxicity, Pt^(IV)^-NP monotherapy failed to induce DSBs in A549^cisR^ cells, as indicated by the low level of γH2AX-positive cells (Fig. 3I and J). Interestingly, exposure of A549^cisR^ cells to NC treatment resulted in a high abundance of γH2AX. The occurrence of DSBs in A549^cisR^ cells was also assessed by a comet assay (Fig. 3K and L). Consistent with the expectation, SN38-NP caused intensive DSBs in cells, and the levels of damaged DNA were markedly increased when cells were treated with the NC. Taken together, these results provide compelling evidence that increased DSBs account for the augmented cytotoxicity, which benefits the combinatorial synergism of the NC regimen.

### The compromised DNA repair capacity was mediated by Rad51 depletion through the ATM/Chk2/p53 pathway

**Fig. 4:**
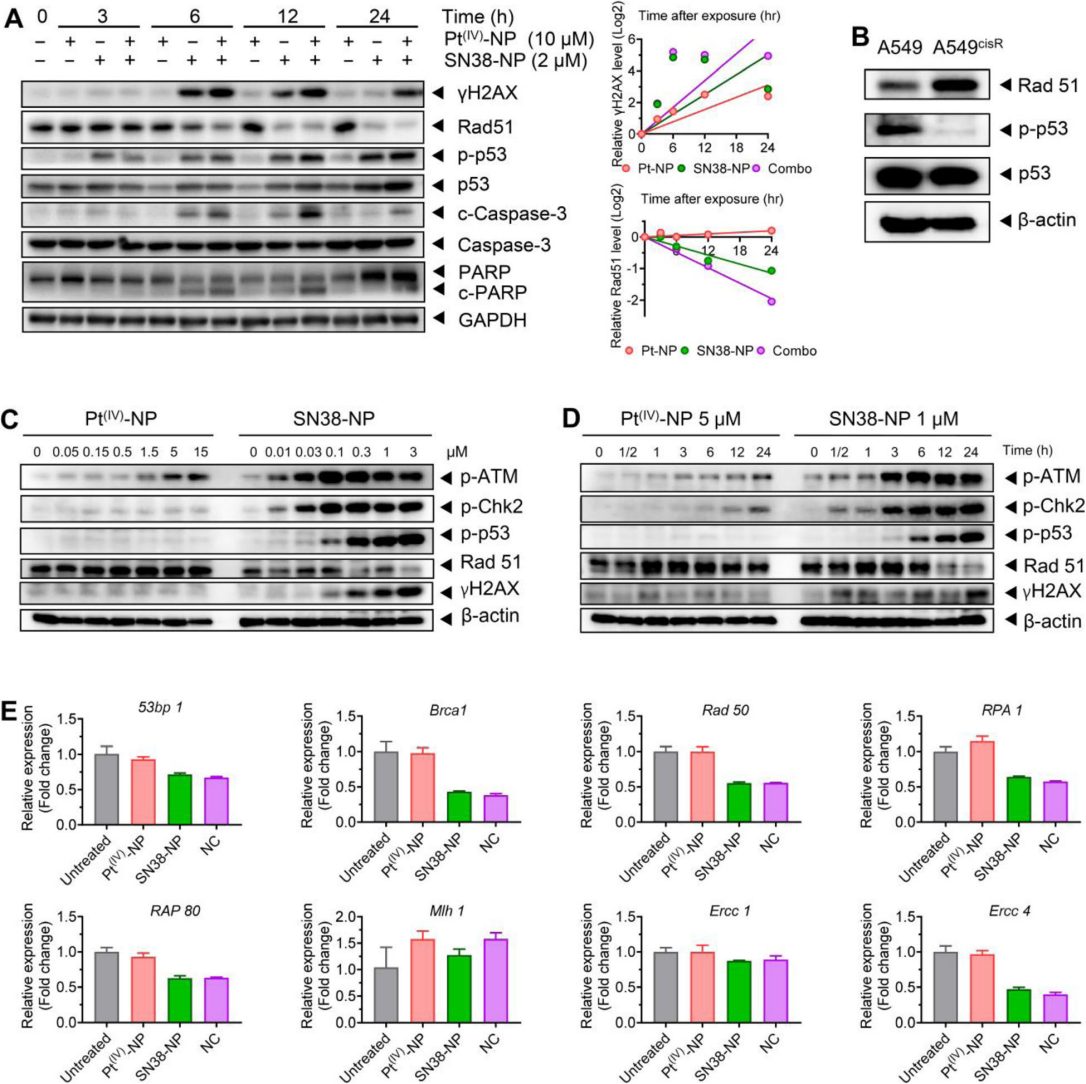
NC compromised the DNA repair capacity of resistant cancer via Rad51 abrogation. (**A**) SN38-NP showed the ability to inhibit DNA repair pathways in resistant cells after Pt^(IV)^-NP treatment. Cells after drug treatment were lysed at the predetermined time points for western blot analysis. Densitometric parameters were quantified by using ImageJ software, and the expression curves are shown (right). (**B**) The expression levels of Rad51, phosphorylated p53 and total p53 in parental A549 and resistant A549^cisR^ cells were examined. (**C** and **D**) The inefficiency of Pt^(IV)^-NP monotherapy in p53 activation and Rad51 inhibition were reversed by SN38-NP through the ATM/Chk2 axis. (**E**) A qPCR assay for the measurement of other key regulators involved in the DNA repair process

Rad51 and its paralogs are known as the crucial components of the homologous recombination (HR) complex machinery involved in the repair of severe DNA damage, such as DSBs [44]. Here, we analyzed the dynamics of Rad51 expression by western blot because its recruitment was the initial event during the DNA damage response. As presented in Fig. 4A, Pt^(IV)^-NP monotherapy considerably increased recruitment of Rad51 to damage sites in A549^cisR^ cells, potentially due to a repair mechanism. Interestingly, the addition of SN38-NP ameliorated Rad51 recruitment, which was also accompanied by a notably high level of γH2AX expression. These results indicate more damaged DNA accumulation in the nuclei. We further observed a negative correlation between the expression of Rad51 and γH2AX, suggesting that DNA lesions induced by cotherapy overrode the repair machinery [45–47]. Consequently, cotreatment with the two nanoparticles provoked robust apoptosis, evidenced by elevated levels of proapoptotic proteins (i.e., cleaved caspase 3 and cleaved PARP). These data were in accordance with the apoptotic results by flow cytometry analysis (Fig. 3G).

Published studies have elucidated that the tumor suppressor protein p53 is also involved in the regulation of HR repair, in addition to its role in promoting apoptosis [48]. As expected, upon NC treatment, phosphorylated and total p53 were increased (Fig. 4A). Moreover, Rad51 overexpression and p53 inactivation are responsible for the drug-resistance phenotype [49, 50]. Differences were indeed observed between A549 and A549^cisR^ cells (Fig. 4B). p53 is a transcriptional repressor of Rad51 that is able to disturb Rad51 expression by binding to promoter regions [51]. Additionally, phosphorylated p53 was negatively correlated with Rad51 expression, revealing the existence of a suppressive effect of p53 on Rad51 (Fig. 4A). Thus, SN38-NP is capable of combating platinum-induced chemoresistance through downregulation of the repair mechanism and upregulation of p53.

In the context of exogenous DNA inflictions, the initial phase of the p53 response is primarily orchestrated by the action of ataxia telangiectasia mutated (ATM) kinase, which senses damaged DNA and transmits signals to downstream effectors via phosphorylation [52]. ATM is reported to phosphorylate p53 on multiple sites, with Ser15 as the primary phosphorylation residue [53]. In addition to direct action, ATM can also activate p53 via phosphorylation of checkpoint kinase 2 (Chk2) and other relevant downstream proteins [54–56]. Here, we speculate that the regulatory ATM/Chk2/p53 circuitry might be responsible for the synergistic effect of NC therapy. To explore this possibility, we investigated the status of these proteins in A549^cisR^ cells upon treatment with varying concentrations of nanoparticles. The western blot results showed that Pt^(IV)^-NP alone did not significantly activate ATM, Chk2, or their downstream p53, which was also accompanied by upregulated Rad51 and silencing γH2AX (Fig. 4C). In contrast, relatively low concentrations of SN38-NP dramatically increased ATM and Chk2 phosphorylation, thereby leading to substantial activation of p53 with less Rad51 expression. Similar trends were observed in cells treated for different durations, where p53 upregulation was positively correlated with ATM and Chk2 activation but was inversely associated with Rad51 expression after SN38-NP treatment (Fig. 4D). Furthermore, we examined the expression levels of other key DNA repair proteins (Fig. 4E). Several of them were also moderately inhibited by cotreatment, although the potential regulatory mechanism remains unclear. Collectively, these data indicate that SN38 sensitizes Pt^(IV)^ agents delivered in single nanotherapy by triggering DNA damage effectors (e.g., enhanced phosphorylation of H2AX and ATM/Chk2) while simultaneously impairing the repair mechanism (e.g., Rad51).

### In vivo activity in a preclinical NSCLC mouse model

**Fig. 5:**
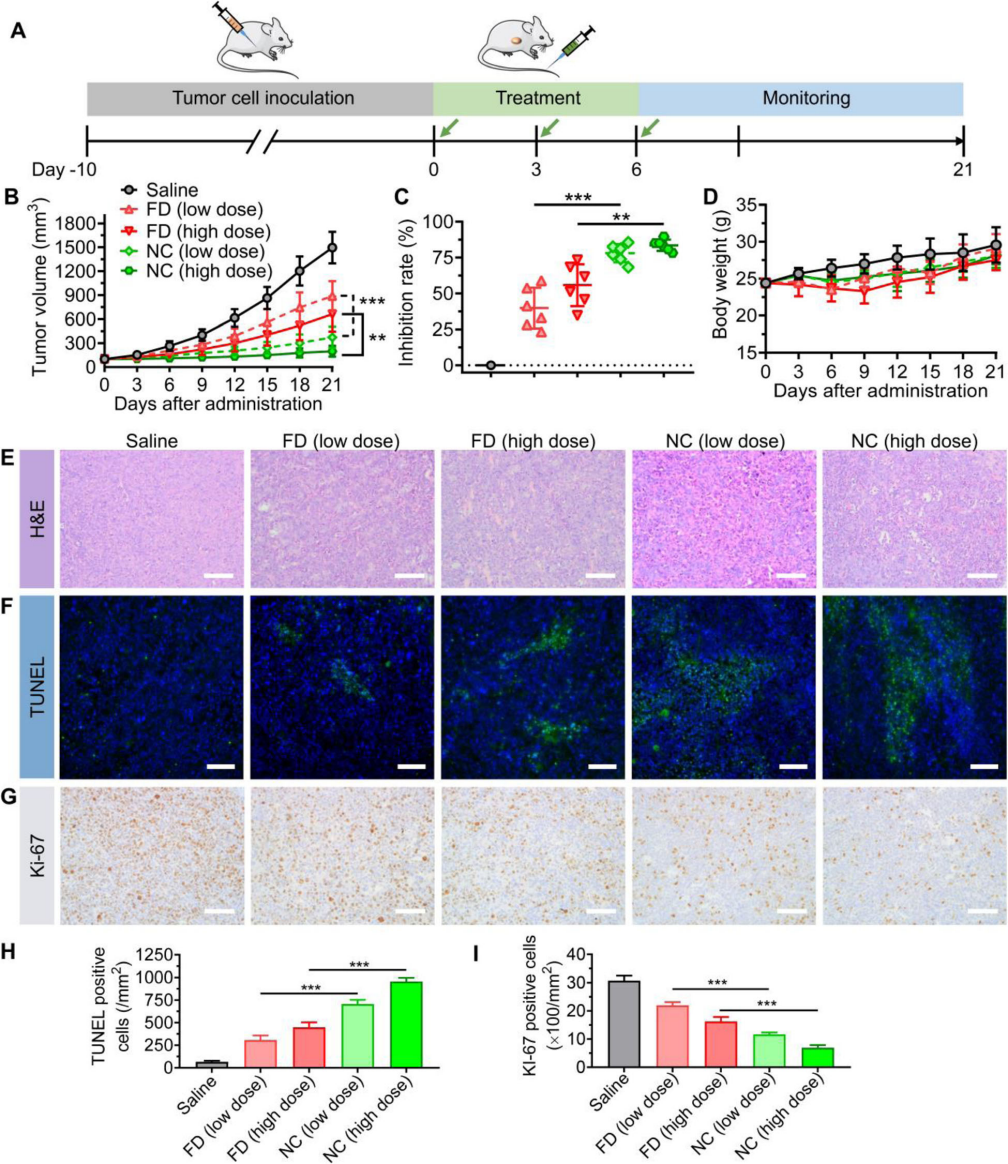
Comparative efficacy study of the NC versus free drug combination (FDC) regimens in a preclinical human lung cancer mouse model. (**A**) Experimental schedule elucidating the establishment of the A549^cisR^ xenograft model and drug administration. (**B**) The change in tumor volume was recorded using a Vernier caliper. Mice were treated with various drug combinations. (**C**) The tumor inhibition rate of each treatment is presented. (**D**) Variances in the bodyweight of each grouped mouse were measured after the treatment**.** (**E-G**) Histological assessment was used to evaluate the therapeutic effects. The excised tumors were harvested and histologically analyzed. Scale bars, 50 μm. (**H** and **I**) The quantification of TUNEL-positive (H) and Ki-67-positive (I) cells in each group is shown in the histogram

In cisplatin-resistant A549^cisR^ cells, combinatorial therapy incurs a high incidence of DSB catastrophe with a low repair response. Inspired by this finding, we examined the potential of NC therapy in a preclinical mouse model bearing A549^cisR^ tumor xenografts (Fig. 5A). The model was established in immunodeficient BALB/c nude mice by subcutaneous implantation of A549^cisR^ cancer cells. When the tumor volume reached approximately 100 mm^3^, mice were randomly divided into five groups and intravenously injected with various drugs. As shown in Fig. 5B, the saline-treated mice exhibited rapid tumor growth, which was ~ 15-fold larger than the initial tumor volume at the endpoint. However, A549^cisR^ tumors only showed moderate responses to treatment with free drug combinations. The NC treatments significantly outperformed the free drug combinations to reduce the tumor burden. Notably, the high dose of NC therapy resulted in durable tumor recession and lasting efficacy during the observation. Consistent results were obtained through analysis of the tumor inhibition rate (Fig. 5C). In addition, the NC therapies had no noticeable toxicity, whereas administration of free drugs resulted in body weight loss (Fig. 5D).

Histopathological analyses of the excised tumors were further performed. Consistent with the growth kinetics, extensive intratumoral apoptosis was induced by NC therapy, as evidenced by hematoxylin and eosin (H&E) and TUNEL staining (Fig. 5E and F). Quantification of TUNEL-positive cells verified the increased apoptotic percentages after NC treatment (Fig. 5H). Concomitantly, IHC staining with the indicator Ki-67 validated the retarded cell proliferation in sectioned tumors after NC therapy (Fig. 5G and I). The in vivo results provide evidence that a combination nanoparticle regimen that encapsulates PUFAylated prodrugs at optimal drug ratios proved effective in eliminating resistant cancer and was superior to the combination of free drugs in their clinical formulations.

### Safety evaluation of the combinatorial NC

**Fig. 6:**
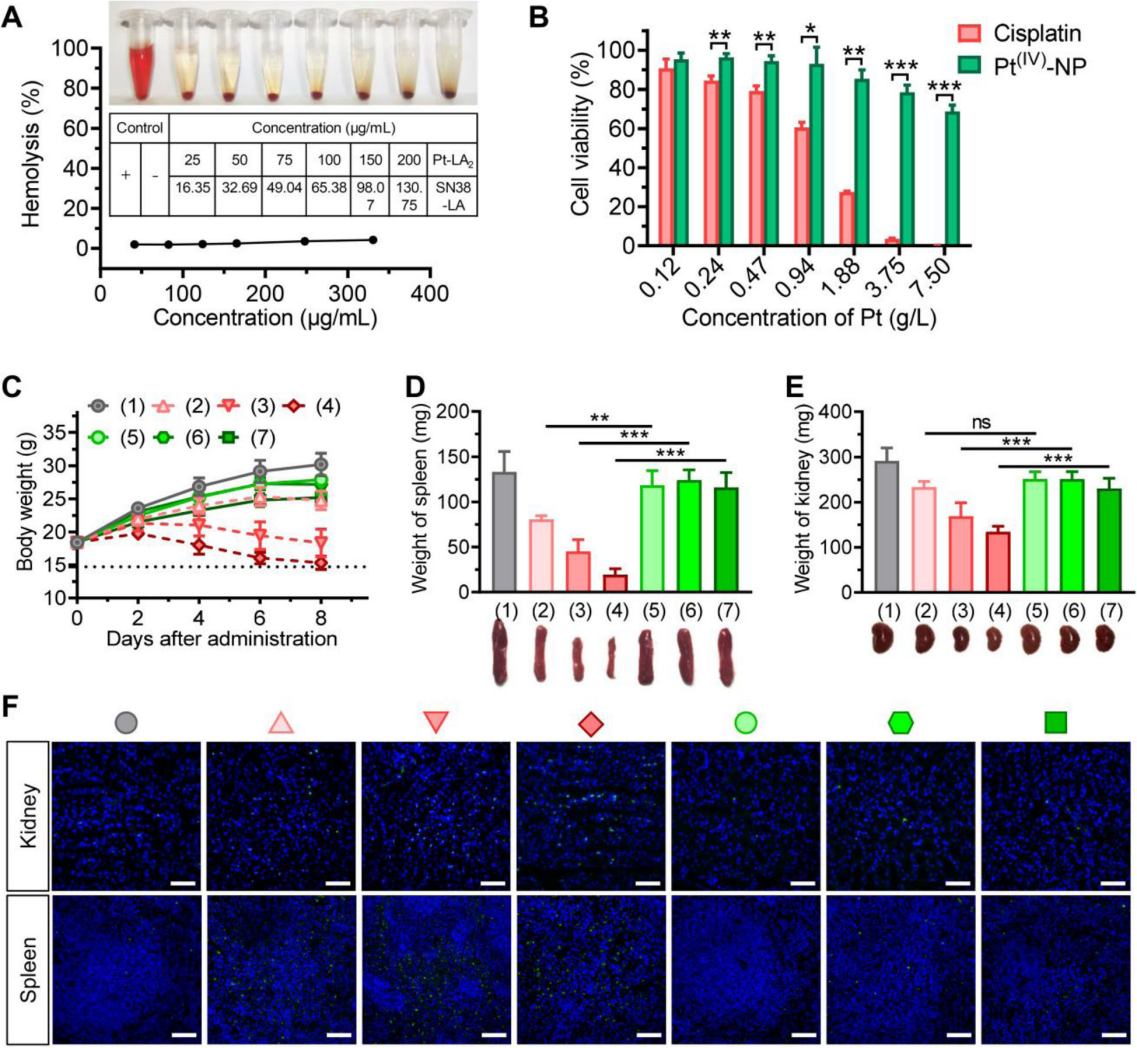
Safety evaluation of NC therapy in comparison to their corresponding clinical formulations in healthy ICR mice. (**A**) The percentage of RBC hemolysis upon varying concentrations of the NC combinations was normalized to that with the positive control (Triton X-100). (**B**) Cytotoxicity of Pt^(IV)^-NP and cisplatin in noncancerous BEAS-2B cells. (**C**) Bodyweight change was monitored during the observation after four injections of drugs. Healthy mice were intraperitoneally administered (1) saline; (2) FD combination (cisplatin, 3 mg/kg plus CPT-11, 2.9 mg/kg); (3) FD combination (cisplatin, 5 mg/kg plus CPT-11, 4.9 mg/kg); (4) FD combination (cisplatin, 8 mg/kg plus CPT-11, 7.8 mg/kg); (5) NC (3 mg/kg platinum equivalence plus 2 mg/kg SN38 equivalence); (6) NC (5 mg/kg platinum equivalence plus 3.3 mg/kg SN38 equivalence); and (7) NC (8 mg/kg platinum equivalence plus 5.3 mg/kg SN38 equivalence). The data are presented as the means ± SD (*n* = 6). (**D**-**F**) The spleen (**D**) and kidney (**E**) were excised and weighed and subsequently subjected to TUNEL staining (**F**) on Day 2 after the last administration. Scale bars, 50 μm

Despite the excellent in vivo antitumor efficacy, the tolerability of nanomedicines in vivo is another indispensable factor when considering clinical translation. In clinical practice, hemolysis was observed in cancer patients receiving excipients, as these materials interacted with erythrocytes [57]. We thus first conducted a hemolytic test to evaluate the blood compatibility of our NC system. Upon exposure of rat blood cells (RBCs) to different concentrations of the NC therapies, only limited hemolysis (2–5.6%) was observed, indicating acceptable low hemolytic activity for intravenous administration (Fig. 6A). Moreover, Pt^(IV)^ prodrug-assembled nanoparticles displayed less cytotoxicity against BEAS-2B normal lung epithelial cells than the unmodified cisplatin agent (Fig. 6B).

Finally, we investigated the in vivo safety of the combination NC regimen. Because cisplatin is more toxic than CPT-11 or SN38 in vivo, we chose the NC platform prepared with a platinum/SN38 drug molar ratio of 2:1 for toxicity studies. NC therapy at this ratio still retained synergism, as shown in the CI values. After the drugs were injected into healthy ICR mice, the variation in body weights was monitored. All NC-treated mice showed a negligible drop in body weight, whereas the mice dosed with FD combinations at high doses (e.g., 5 mg/kg cisplatin plus 4.9 mg/kg CPT-11) exhibited a remarkable decline in body weight (Fig. 6C). In addition, nephrotoxicity remains the dose-limiting toxicity for cisplatin chemotherapy. Obvious nephrotoxicity and hematotoxicity in the mice receiving the FD combination regimens were observed. Moreover, the necropsy results revealed significant reductions in the weights of the kidney and spleen compared with those receiving the NC and saline treatments (Fig. 6D and E). To further explore the underlying mechanism of the observed visceral toxicity, we subjected the sectioned kidney and spleen to histological analysis. TUNEL staining validated that both tissues excised from the FD-treated mice suffered significant apoptosis, which contrasted sharply with undetectable apoptosis in the organs from the nanoparticle-treated mice (Fig. 6F). No apparent pathological changes were found in other organs (e.g., heart, liver, and lung) (Fig. S13). These toxicity studies demonstrate that coassembled nanococktails from rationally designed prodrugs are sufficiently safe for preclinical cancer treatment.

## Discussion

Lung cancer is the second most common cancer and the leading cause of cancer-related death worldwide, with an estimated 2.2 million new cases and 1.8 million new deaths in 2020 [58]. NSCLC is the major subtype (~ 85%) of lung cancer, with a very poor overall 5-year survival rate of approximately 15% [59]. In the clinic, platinum-based chemotherapies such as cisplatin are extensively used for the treatment of NSCLC patients. Platinum drugs mainly perturb DNA synthesis via the formation of covalent DNA-Pt adducts between adjacent purine residues such as guanine, leading to cancer cell apoptosis [60]. However, repeated administration of platinum agents is associated with dose-limiting toxicities and drug resistance. The mechanisms of cisplatin resistance are multifactorial, including reduced drug uptake by alteration of membrane transporters, detoxified platination by metabolic enzymes, upregulation of cell survival signals, and enhanced DNA repair [31, 61, 62]. Thus, combining cisplatin with other agents harboring nonoverlapping cytotoxic mechanisms seems to be an attractive therapeutic paradigm for handling drug resistance.

SN38, the active form of CPT-11, under the trademark of irinotecan, is a potent TOP1 inhibitor against a variety of human malignant cancers. Unfortunately, SN38 is either water-insoluble or has low compatibility for encapsulation in conventional delivery carriers [63]. Mechanistically, after exposed to rapidly proliferating tumor cells, this camptothecin agent noncovalently binds to nuclear DNA TOP1 and generates replication-mediated DSBs. Unlike camptothecin poison, platinum agents typically form intra- and interstrand covalent DNA cross-links with purine nucleobases. These cross-links impair transcription and lead to cell apoptosis. Due to the different mechanisms of action, both therapeutics are expected to have a complementary role in inducing DNA damage and causing elevated cell apoptosis against resistant cancer cells. However, owing to the large dissimilarity in physicochemical properties, simultaneous engineering of these two compounds into a single platform is technically challenging.

To address this unmet medical need, we engineered platinum (II) and SN38 derivatives using our previously established PUFAylation strategy. Due to their structural similarity, the resultant Pt-LA_2_ and SN38-LA prodrug entities are miscible in each other at varying drug ratios for the production of injectable synergistic cocktails in aqueous solutions. The use of naturally occurring PUFAs for drug derivatization and subsequent pharmaceutical delivery via nanoassembly has particular advantages: (i) PUFAs are commercially available and can be readily accessible in large quantities; (ii) they are abundant in the body and are essential for humans, which avoids the risks associated with excipient side effects; and (iii) potential π − π stacking and hydrophobic interactions between PUFAs could make nanoassemblies sufficiently stable for systemic injection. More interestingly, drug ratios in this coassembling system can be fine-tuned to yield high antitumor synergy with reduced toxicity. These advantages could make PUFAs promising as useful skeletons for the generation of clinically translatable nanoassemblies.

In vitro cytotoxicity studies revealed a significantly high synergistic effect (CI ~ 0.271) of the NC against A549^cisR^ cells when the NC was prepared with a molar ratio of Pt-LA_2_ to SN38-LA of 5:1. Of note, Pt^(IV)^-NP assembled from the Pt-LA_2_ prodrug outperformed cisplatin in overcoming drug resistance. For example, Pt^(IV)^-NP showed a 4-fold higher antiproliferative potency than cisplatin in A549^cisR^ cells. Subsequent CLSM observation confirmed that the internalization of the NC platform relies on an endocytosis pathway and is independent of the Ctr1 transporter that is necessary for cellular cisplatin uptake. The synergistic effects of the NC were further validated with cell-based analyses. The EdU assay, together with the cell cycle analysis, showed that the NC treatment retarded the cell growth of the resistant cancer cells by arresting more cells in S and G_2_ phases compared to individual nanotherapies. Moreover, exposure of cancer cells to the NC resulted in elevated levels of apoptotic cells, which was accompanied by excessive DNA lesions, as unveiled by γH2AX staining and comet assay.

**Fig. 7:**
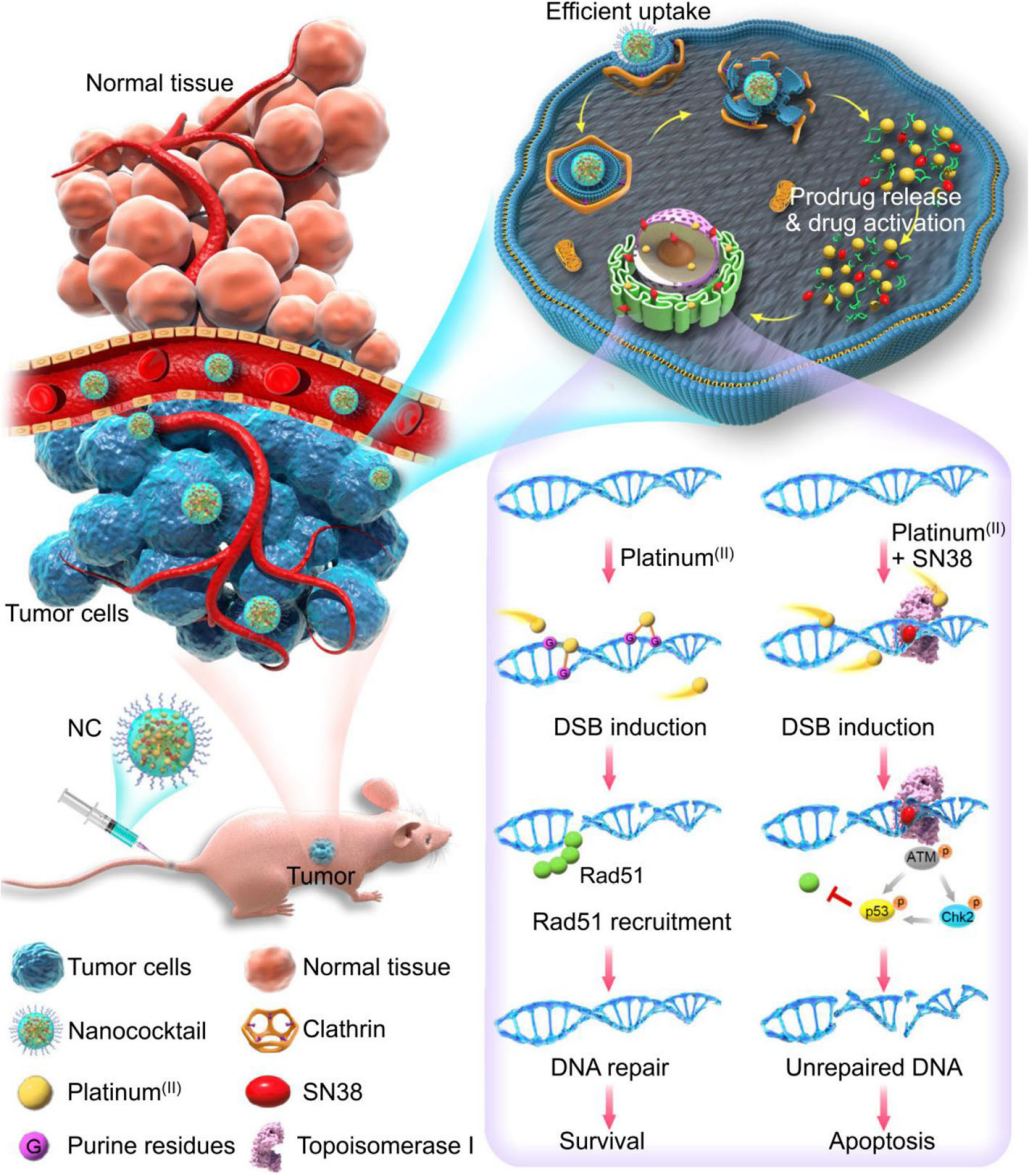
Schematic of the nanococktail assembled from small-molecule prodrugs for intratumoral delivery and intranuclear synergistic mechanism to overcome chemoresistance. After intravenous administration, NC extravasates into the tumor and can be effectively internalized by cancer cells. Therapeutically active drugs can be released from the NC and enter the nucleus to poison DNA. Platinum (II) monotherapy is inefficient to cause DSBs due to Rad51-mediated DNA repair. Additional SN38 therapy is anticipated to induce extensive DSBs and provoke robust apoptosis by depleting the repair protein through the ATM/Chk2/p53 axis, leading to optimal efficacy against drug-resistant NSCLC

Previous studies have demonstrated that Rad51 plays a crucial role in HR-mediated DSB repair and is activated in platinum-resistant cell lines [64]. Cellular investigation showed that Rad51 recruitment occurred after Pt^(IV)^-NP exposure, indicating the existence of DSB repair machinery, which compromised the efficacy of Pt^(IV)^-NP monotherapy. Unexpectedly, we observed that Rad51 recruitment was markedly inhibited when the cells were further poisoned with SN38-NP (Fig. 4A, C, and D). These data suggest that the SN38 agent has a role in inhibiting Pt^(IV)^-NP-mediated DSB repair. We also confirmed that the NC treatment resulted in reduced Rad51 expression and augmented DSBs as evidenced by elevated levels of γH2AX in A549^cisR^ cells, which caused potent apoptotic signaling cascades. Further mechanistic studies revealed that Rad51 inhibition was accomplished through the activation of ATM/Chk2/p53 signaling by the camptothecin agent delivered in the NC platform (Fig. 7).

Finally, the in vivo efficacy of the nanococktail was examined in a resistant tumor-bearing mouse model. Administration of NC outperformed free drug-based combinations in slowing aggressive tumor growth. The nanococktail, via coassembly of PUFAylated cisplatin and SN38 prodrugs at the optimal ratio, had superiority over free drugs in effectively eradicating resistant cancer, which could be attributed to the synergism between the two agents as well as accelerated cellular uptake of nanoparticles. Moreover, the small molecular nanosystem was highly biocompatible for in vivo administration, as evidenced by the ameliorated nephrotoxicity and hematotoxicity in comparison to the combination of free drugs in the conventional formulations.

## Conclusion

In summary, distinct from the strategy of using complex nanostructures to incorporate multiple agents, our prodrug coassembling approach provides a facile and cost-effective method for drug codelivery and synergistic cancer therapy. The nanococktail formulating synergistic prodrugs at designated ratios have the potential to thwart resistance in NSCLC and shows low systemic toxicity. In addition, because of the simplicity of prodrug synthesis and nanoparticle manufacture, these small molecular nanoassemblies may have high translational capacity. Further studies are warranted to investigate the long-term safety and therapeutic outcomes.

## Materials and methods

### Cell lines and reagents

Human NSCLC A549 and cisplatin-resistant A549^cisR^ cells were purchased from the Bogu Biotechnology Company (Shanghai, China) and cultured in RPMI-1640 media (Biological Industries, Israel) supplemented with 10% FBS (Biological Industries) and 1% penicillin/streptomycin (Biological Industries) in a humidified incubator containing 5% CO_2_ at 37 °C. For A549^cisR^ cells, an additional 800 ng/mL cisplatin was added to the media to maintain resistance.

Cisplatin and linoleic anhydride were purchased from Tokyo Chemical Industry Co. (Shanghai, China). 7-Ethyl-10-hydroxycamptothecin (SN38) was purchased from Knowshine Pharmachemicals Inc. (Shanghai, China). All other compounds and solvents were purchased from J&K Chemical (Shanghai, China).

### Preparation of Pt-LA_2_ and SN38-LA prodrug nanoparticles

Self-assembled nanoparticles were prepared using a reprecipitation method. A mixture of Pt-LA_2_ and/or SN38-LA conjugates with DSPE-PEG_2k_ at a weight ratio of 10:1 was used to facilitate the preparation. Briefly, mixtures of prodrug/DSPE-PEG_2k_ or prodrug cocktail/DSPE-PEG_2k_ (2 mg, cisplatin or SN38 equivalence) were dissolved in DMSO (1 mL), and then the solutions were injected into 9 mL of DI water under ultrasonication. Prior to subsequent use, the remaining organic solvent was removed by dialysis against DI water.

### Animal experiments

Immunodeficient male BALB/c nude mice (6 weeks old) were purchased from the Shanghai Experimental Animal Center, Chinese Academy of Science. All animal studies were conducted in accordance with the National Institute Guide for the Care and Use of Laboratory Animals. The experimental protocols were approved by the Ethics Committee of the First Affiliated Hospital, Zhejiang University School of Medicine.

### In vivo tumoricidal effect of combinatorial NC therapy

The antitumor activity was evaluated in BALB/c nude mice. Briefly, A549^cisR^ cells (3 × 10^6^ cells/0.1 mL PBS) were subcutaneously injected into the flank of each mouse. When the tumor volume approached approximately 100 mm^3^, the mice were randomized into five groups (*n* = 6) and treated with various drugs as follows: i) saline; ii) free drug (FD) combination (cisplatin, 3 mg/kg plus CPT-11, 2.9 mg/kg (low dose, LD)); iii) FD combination (cisplatin, 5 mg/kg plus CPT-11, 4.9 mg/kg (high dose, HD); iv) the LD nanococktail regimen (3 mg/kg platinum equivalence plus 2 mg/kg SN38 equivalence); and v) the HD NC regimen (5 mg/kg platinum equivalence plus 3.3 mg/kg SN38 equivalence). All treatments were intravenously injected via the tail vein on Days 0, 3, and 6. Tumor growth was recorded by measuring the width (W) and length (L) of the tumors with a caliper for 3 weeks, and body weight was measured to monitor drug toxicity. The tumor volume was calculated using the following formula: tumor volume (mm^3^) = (W)^2^ × (L)/2.

To further assess the antitumor activity, tumor tissues from each treatment group were obtained and sectioned. A terminal deoxynucleotidyl transferase dUTP nick end labeling (TUNEL) assay was carried out using a TdT-Frag EL DNA fragmentation detection kit to analyze apoptotic cell death. H&E staining of the tumor sections was also performed. To visualize the proliferating tumor cells, immunohistochemical staining with antibodies against Ki-67 was performed. All tumor slices were observed and captured using a microscope (IX73, Olympus) at 200× magnification. Five fields per slice were randomly chosen and imaged for analysis.

### Hemocompatibility analysis and toxicity assessment

The hemolysis assay was performed at pH 7.4 using RBCs. RBCs were isolated from plasma by centrifuging at 1500 rpm for 15 min and purified by repeated washes until the supernatant became transparent. RBCs were then resuspended in saline and blended with aliquots of saline containing various concentrations of the NCs. DI water was used as a positive control, with saline treatment serving as the negative control. All samples were gently shaken and deposited at room temperature for 2 h, followed by centrifugation at 1500 rpm for 15 min. The supernatant of each sample was added to a 96-well plate for absorbance measurement at 540 nm. The hemolysis rate was calculated by the following equation: hemolysis (%) = (A_sample_-A_negative control_)/(A_positive control_-A_negative control_) × 100%.

To exactly reflect the toxicity difference between the free drug combinations and combinatorial NC therapy, male ICR mice were intraperitoneally administered various free drugs and nanoformulations. The injection was performed every other day four times. The bodyweight of the mice was monitored every other day to assess systemic toxicity throughout the treatment. At the endpoint, mice were humanely sacrificed, and major organs were collected and subjected to pathological observation to examine drug-induced lesions. Additionally, kidneys and spleens were weighed and photographed because of their vulnerability to platinum-based therapy. Cell apoptosis in vital organs was measured using the TUNEL assay.

### Statistical analysis

All determinations were independently repeated three times, and values are presented as the means ± SD. Statistical analysis was performed in Prism 8.0 software, where a two-tailed Student’s *t*-test was conducted to compare the significant difference between two groups, and a one-way ANOVA test was used to analyze the differences among more than three groups. Differences with *p* values less than 0.05, 0.01, and 0.001 were considered to indicate statistical significance at different probability levels and marked with *, **, and ***, respectively.

## Supplementary Information

**Additional file 1.** Supporting Information. Combinatorial nanococktails via self-assembling lipid prodrugs for synergistically overcoming drug resistance and effective cancer therapy.

## Data Availability

The datasets used and/or analyzed during the current study are available from the corresponding authors on reasonable request.

## Abbreviations

PUFAs: Polyunsaturated fatty acids
NSCLC: Non-small cell lung cancer
NC: Nanococktail
SN38: 7-ethyl-10-hydroxy-camptothecin
LA: Linoleic acid
DSPE-PEG2k: 1,2-distearoyl-sn-glycero-3-phosphoethanolamine-N-[methoxy-(polyethyleneglycol)2000]
CI: Combination index
MPS: Mononuclear phagocyte system
DMSO: Dimethyl sulfoxide
TEM: Transmission electron microscopy
DLS: Dynamic light scattering
PDI: Polydispersity index
PBS: Phosphate-buffered saline
Ctr1: Copper transport protein 1
TOP1: Topoisomerase I
IC_50_: Half-maximal inhibitory concentrations
CLSM: Confocal laser scanning microscope
EdU: 5-ethynyl-2′-deoxyuridine
γH2AX: H2A histone family member X
ATM: Ataxia telangiectasia mutated
Chk2: Checkpoint kinase 2
H&E: Hematoxylin and eosin
RBCs: Rat blood cells
