# Supplementary material for: Combinatorial nanococktails via self-assembling lipid prodrugs for synergistically overcoming drug resistance and effective cancer therapy
Source: Biomater Res. 2022 Jan 31;26:3. doi: 10.1186/s40824-022-00249-7 (PMC8805243; doi:10.1186/s40824-022-00249-7)
Supplement: Supplementary file 1 — Additional file 1. Supporting Information. Combinatorial nanococktails via self-assembling lipid prodrugs for synergistically overcoming drug resistance and effective cancer therapy. [file 40824_2022_249_MOESM1_ESM.pdf]

Supporting Information

Combinatorial nanococktails *via* self-assembling lipid prodrugs for synergistically overcoming drug resistance and effective cancer therapy

Tongyu Li,[1,#] Weiwei Shi,[2,#] Jie Yao,[1,3] Jingyun Hu,[4] Qiong Sun,[2] Jing Meng,[2] Jian Wan,[5,] Haihan Song,[4,*] and Hangxiang Wang[1,*]*

[1] The First Affiliated Hospital, Zhejiang University School of Medicine; NHC Key Laboratory of Combined Multi-Organ Transplantation; Key Laboratory of Organ Transplantation, Research Center for Diagnosis and Treatment of Hepatobiliary Diseases, Zhejiang Province, Hangzhou, PR China

[2] Department of Oncology, The First Medical Center, Chinese PLA General Hospital, Beijing, PR China

[3] Department of Chemical Engineering, Zhejiang University, Zhejiang Province, Hangzhou, PR China

[4] Central Lab, Shanghai Key Laboratory of Pathogenic Fungi Medical Testing, Shanghai Pudong New Area People's Hospital, Shanghai, PR China

[5] Department of Emergency and Critical Care Medicine, Shanghai Pudong New Area People's Hospital, Shanghai, PR China

Corresponding Authors:

***Jian Wan**, Department of Emergency and Critical Care Medicine, Shanghai Pudong New Area People's Hospital, Shanghai, China. Email: drjian@yeah.net*

***Haihan Song**, Central Lab, Shanghai Key Laboratory of Pathogenic Fungi Medical Testing, Shanghai Pudong New Area People's Hospital, Shanghai, China. Email: haihansong@163.com*

***Hangxiang Wang**, The First Affiliated Hospital, Zhejiang University School of Medicine; 79, Qingchun Road, Hangzhou 310003, China. E-mail: wanghx@zju.edu.cn*

[#] T.L. and W.S. contributed equally to this work.

Synthesis of Cisplatin-Derived Prodrug

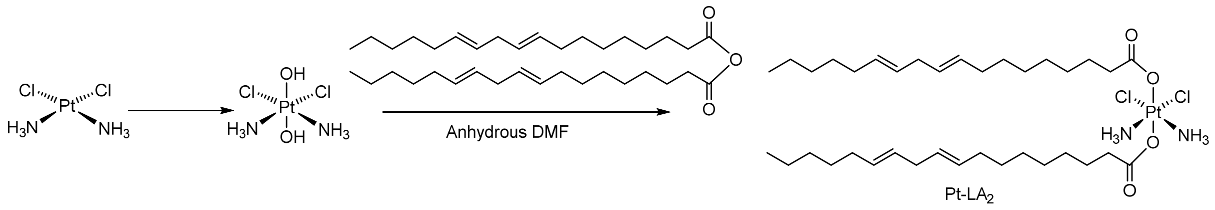

Figure S1. The synthetic procedure of the Pt-LA2 conjugate.

The prodrug of Pt-LA2 was synthesized according to our previous works [1]. In brief, *cis, cis, trans*-$Pt(NH_3)_2Cl_2(OH)_2$ was first synthesized through an oxidation reaction. Cisplatin (1.0 g, 3.3 mmol) was added to a solution of 30% hydrogen peroxide (H_2O_2, 30 mL) in a round-bottomed flask and continuously stirred at 80°C for 8 h in dark. The reaction solution was then stored at 4°C overnight to obtain precipitate, followed by washing with deionized water, ethanol, and ethyl ether. The resulting residue was dried under vacuum to afford pale-yellow solid (0.91 g, 82%).

Next, *cis, cis, trans*-$Pt(NH_3)_2Cl_2(OH)_2$ (200 mg, 0.6 mmol) and linoleic anhydride (814 mg, 1.5 mmol) were dissolved in anhydrous *N, N*-dimethylformamide (DMF, 4 mL) and stirred at 75°C for 6 h. The reaction was monitored by thin-layer chromatography (TLC). The solvent was then removed by vacuum, and the residue was resuspended in dichloromethane (DCM), followed by wash with saturated brine. The organic layer was dried over anhydrous Na_2SO_4, filtered, and evaporated under vacuum. The residue was purified by flash column chromatography on silica gel (DCM: MeOH = 100: 1) to give a yellow solid product (401 mg, 78%).

^{1}H NMR (400 MHz, chloroform-*d*): δ 5.76 (s, 6H), 5.45 – 5.28 (m, 8H), 2.77 (t, J = 6.5 Hz, 4H), 2.45 (t, J = 7.6 Hz, 4H), 2.08 – 1.99 (m, 8H), 1.57 (t, J = 7.4 Hz, 4H), 1.34 – 1.25 (m, 28H), 0.89 (t, J = 6.7 Hz, 6H).

^{13}C NMR (100 MHz, Chloroform-*d*) δ 184.69, 184.69, 130.30, 130.30, 130.02, 130.02, 128.10, 128.10, 127.90, 127.90, 36.37, 36.37, 31.53, 31.53, 29.72, 29.72, 29.72, 29.72, 29.37, 29.37, 29.29, 29.29, 29.29, 29.29, 27.26, 27.26, 27.21, 27.21, 25.99, 25.99, 25.64, 25.64, 22.61, 22.61, 14.13, 14.13.

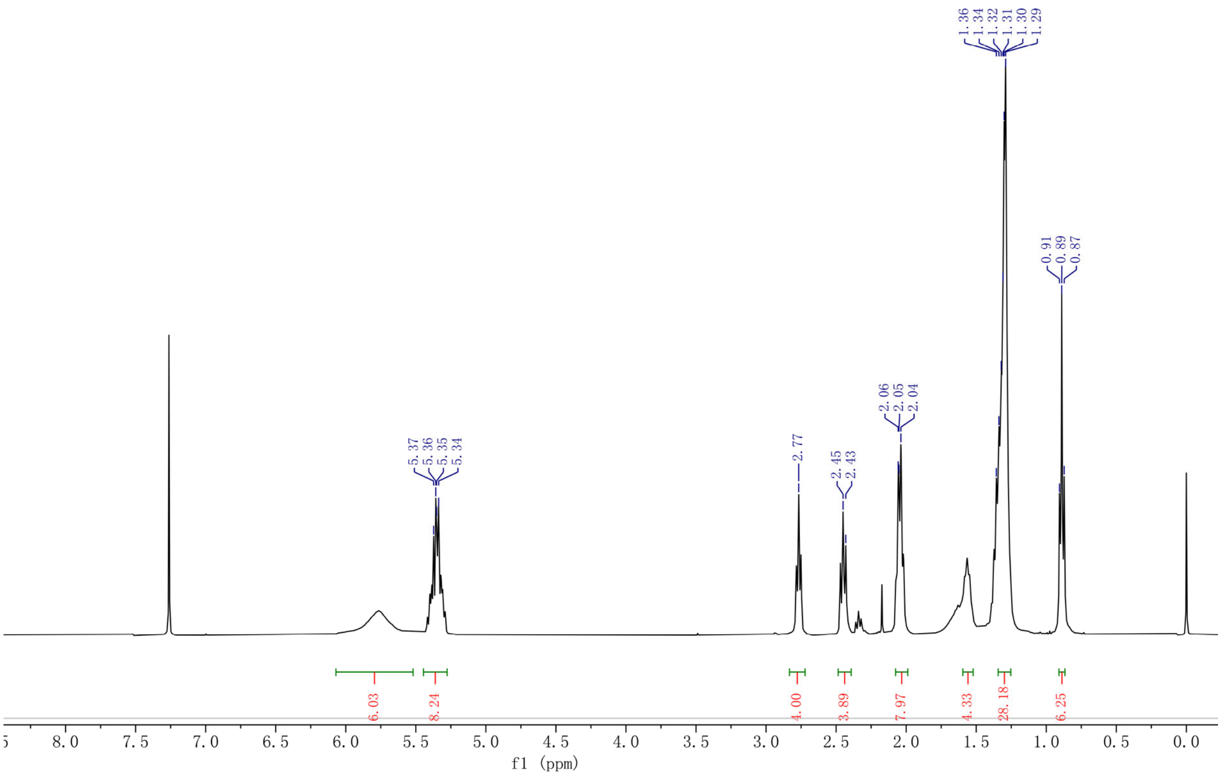

Figure S2. ^{1}H NMR spectrum of the cisplatin-derived Pt-LA_2 prodrug in chloroform-*d*.

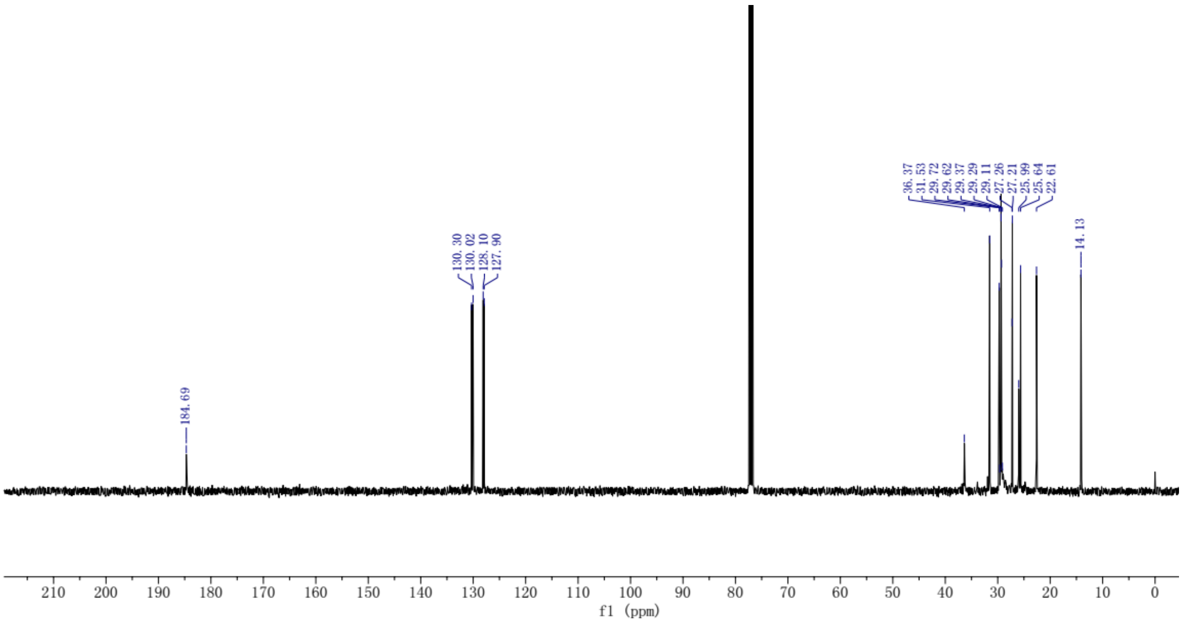

Figure S3. ^{13}C NMR spectrum of the Pt-LA_2 prodrug in chloroform-*d*.

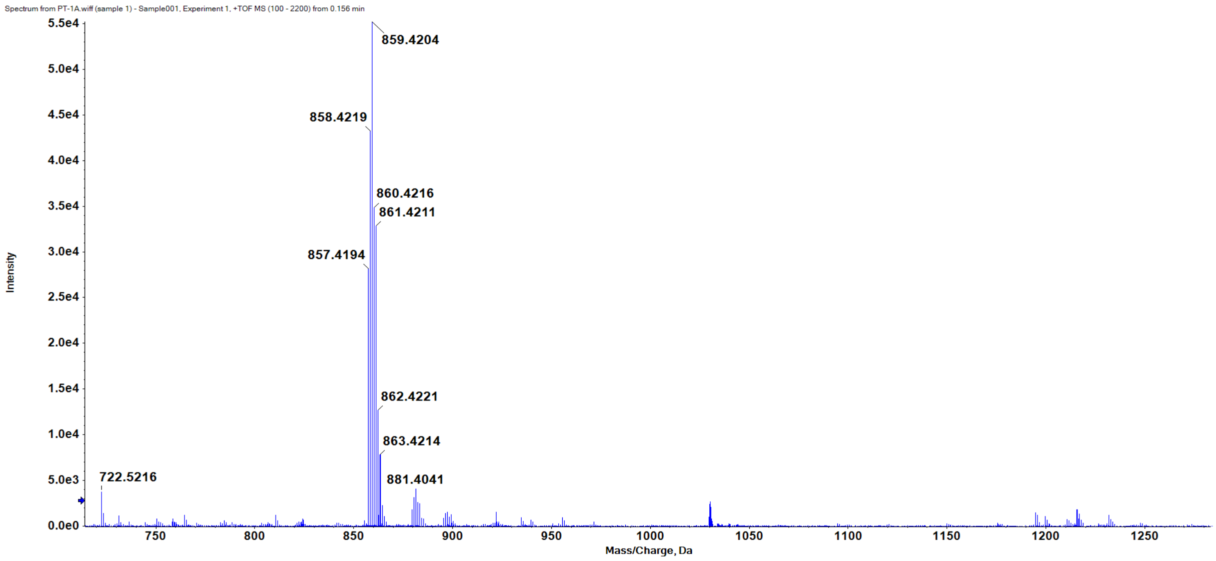

Figure S4. High-resolution mass spectrum of the Pt-LA_2 prodrug.

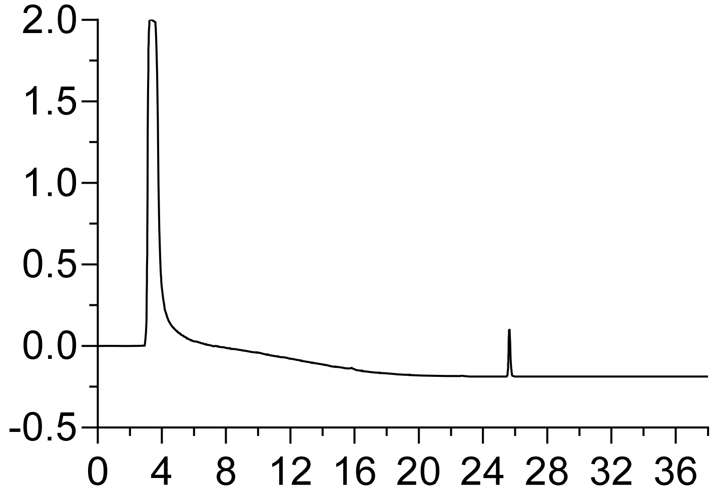

Figure S5. RP-HPLC chromatogram of the Pt-LA_2 prodrug. The prodrug was subjected to analytical HPLC using a C8 reverse-phase column (5 μm, 250 mm × 4.6 mm). A gradient of 30-100% acetonitrile in water within 20 min was adopted as the mobile phase at a flow rate of 1 mL/min together with UV detection at a wavelength of 220 nm.

Synthesis of SN38-Derived Prodrug

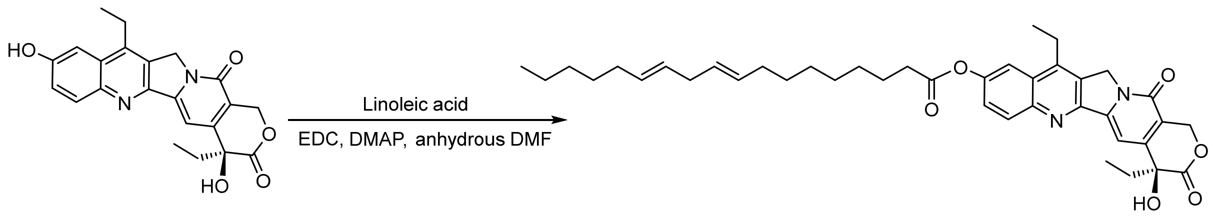

Figure S6. The synthetic procedure of the SN38-LA conjugate.

SN38 (196 mg, 0.5 mmol) was added to a solution of linoleic acid (168 mg, 0.6 mmol), DMAP (73 mg, 0.6 mmol), and EDC (93 mg, 0.6 mmol). The reaction mixture was stirred at 43°C for 2 h. After the solvent was removed, DCM was added and washed with 5% citric acid to remove DMAP, saturated $NaHCO_3$, and brine. The organic layer was dried over anhydrous Na_2SO_4, filtered, and evaporated under vacuum. The crude residue was purified by flash column chromatography on silica gel (ethyl acetate/hexane = 1:3) to give the final compound (265 mg, 82%).

^{1}H NMR (400 MHz, chloroform-*d*): δ 8.24 (d, J = 9.1 Hz, 0H), 7.82 (d, J = 2.5 Hz, 0H), 7.66 (s, 0H), 7.55 (dd, J = 9.1, 2.5 Hz, 0H), 5.76 (d, J = 16.3 Hz, 0H), 5.45 – 5.25 (m, 2H), 3.80 (s, 0H), 3.16 (q, J = 7.6 Hz, 1H), 2.79 (t, J = 6.4 Hz, 1H), 2.67 (t, J = 7.5 Hz, 1H), 2.11 – 2.01 (m, 1H), 1.86 (ddq, J = 29.9, 15.2, 7.4 Hz, 1H), 1.49 – 1.23 (m, 4H), 0.89 (t, J = 6.7 Hz, 1H).

^{13}C NMR (100 MHz, Chloroform-*d*) δ 173.89, 172.25, 157.65, 151.79, 150.25, 149.71, 147.33, 146.82, 145.30, 132.05, 130.29, 130.00, 128.16, 127.89, 127.45, 127.27, 125.50, 118.60, 114.53, 98.16, 72.84, 66.30, 49.42, 34.45, 31.61, 31.55, 29.73, 29.63, 29.37, 29.22, 29.14, 27.23, 27.21, 25.66, 24.86, 23.19, 22.60, 14.13, 14.01, 7.87.

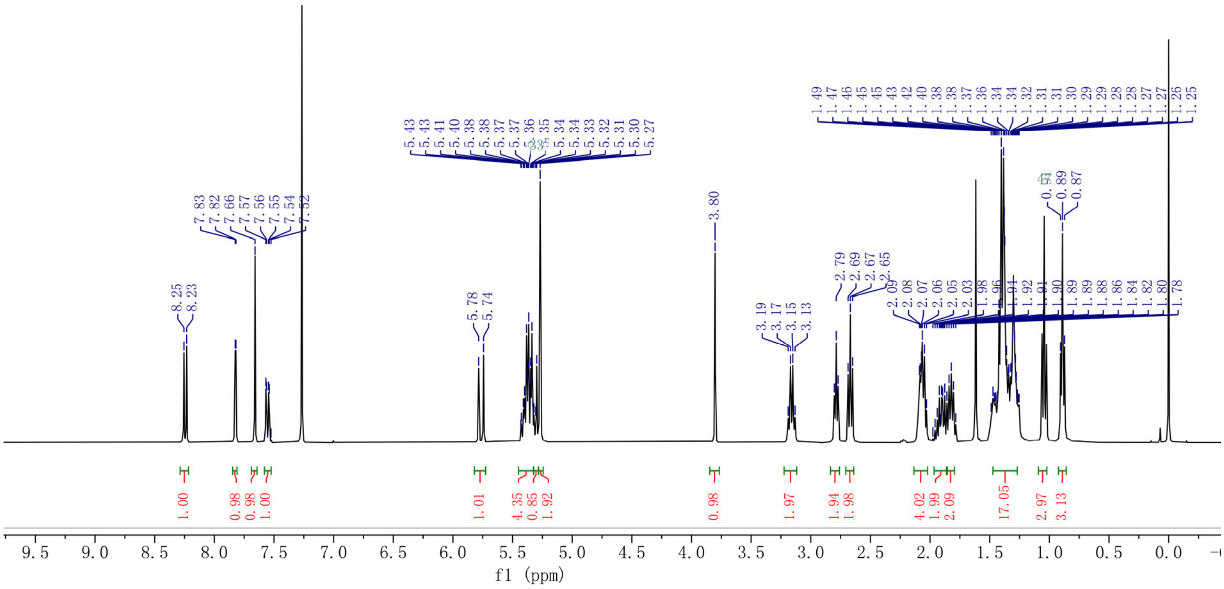

Figure S7. ^{1}H NMR spectrum of the SN38-derived SN38-LA prodrug in chloroform-*d*.

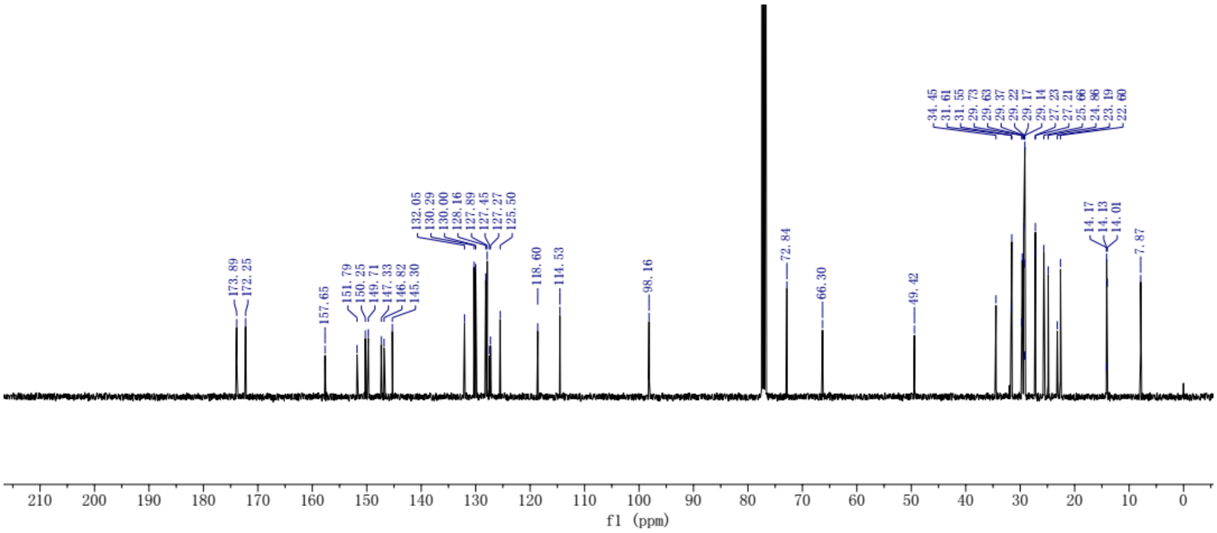

Figure S8. ^{13}C NMR spectrum of the SN38-LA prodrug in chloroform-*d*.

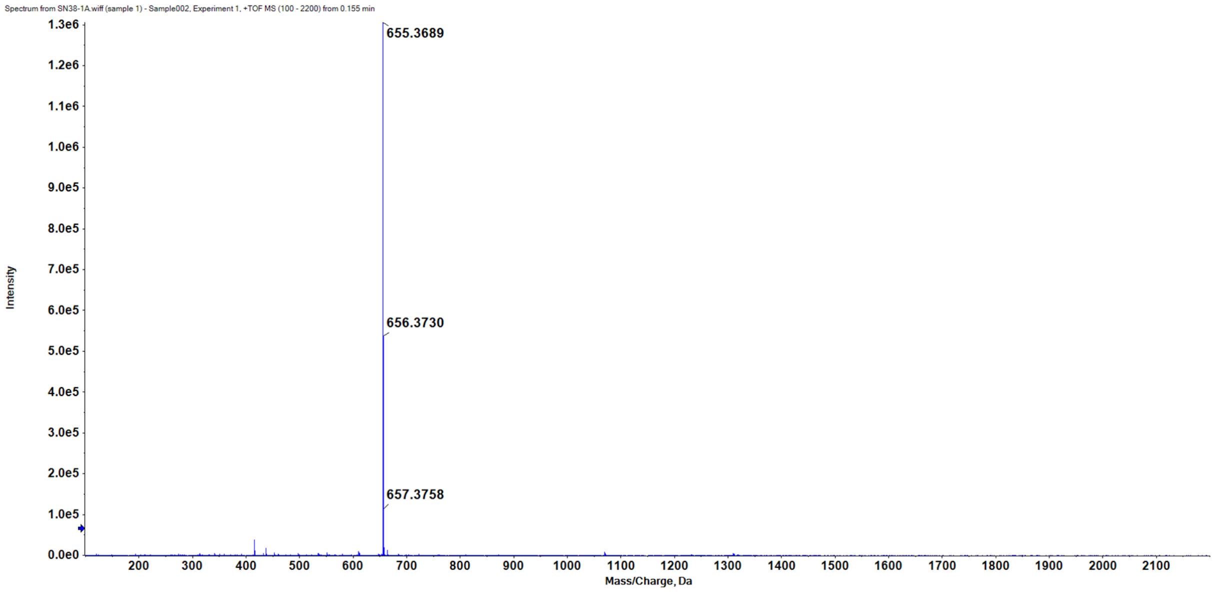

Figure S9. High-resolution mass spectrum of the SN38-LA prodrug.

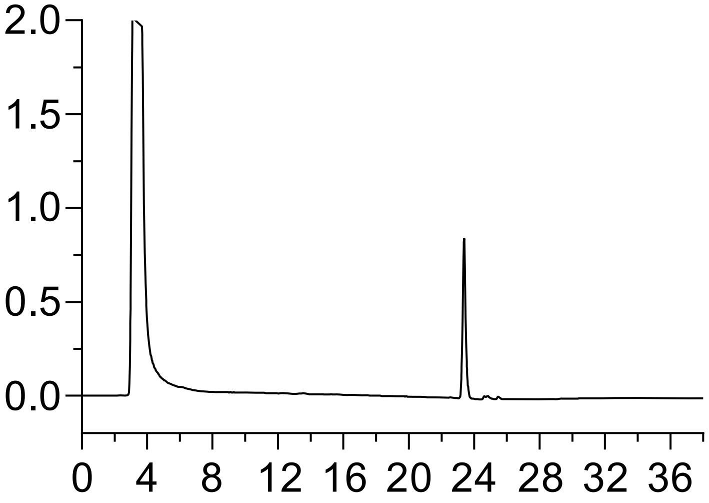

Figure S10. RP-HPLC chromatogram of the SN38-LA prodrug. The prodrug was subjected to analytical HPLC using a C8 reverse-phase column (5 μm, 250 mm × 4.6 mm). A gradient of 30-100% acetonitrile in water within 20 min was adopted as the mobile phase at a flow rate of 1 mL/min together with UV detection at a wavelength of 220 nm.

Size Characterization

The hydrodynamic diameters (D_H), ζ potentials, and polydispersity indexes (PDI) of prodrug-loaded nanoparticles were obtained from dynamic light scattering (DLS) measurement on a Malvern Nano-ZS90 instrument (Malvern, UK) at 37 °C, with each sample repeated thrice.

Transmission Electron Microscopy Analysis

Transmission electron microscopy (TEM) images were captured by using TECNAL 10 (Philips). NPs at a concentration of 0.5 mg/mL (cisplatin and SN38 equivalence) were dripped on a 300-mesh copper grid. After a 2-minute deposition, the surface water was removed with filter paper and then air-dried. The surface of each grid was further positively stained with an aqueous solution of 2 wt % uranyl acetate.

Cell viability assay and analysis of synergistic effects

A total number of 3×10^3 cells of A549 cells and $A549^{cisR}$ cells were seeded in 96-well plates and cultured for 24 h for cell adherence. Next, each cell line was treated with a variety of concentrations of $Pt^{(IV)}$-NP, SN38-NP, or the nanococktails (NCs) at constant molar ratios of 10:1, 5:1, 2:1, 1:1, 1:2, 1:5, or 1:10 for an additional 72 h at 37 °C. Finally, cell viability was measured by a CCK-8 kit according to the manufacturer's protocol. Half-maximal inhibition values were extrapolated from inhibition curves by using Graphpad Prism software (8.0). A combination analysis was performed using the method described by Chou and a Calcusyn software program for automated analysis [2]. The synergy of $Pt^{(IV)}$-NP in combination with SN38-NP was evaluated by calculating the combination index at IC_{50} (termed CI_{50}) using the following equation: $CI_{(50)} = (dose)_1/(dose\ x)_1 + (dose)_2/(dose\ x)_2$, where $(dose\ x)_1$ and $(dose\ x)_2$ represented the separated concentrations of drug 1 and drug 2 that result in (x) % inhibitory effect in cell viability and $(dose)_1$ and $(dose)_2$ were the concentrations of drug 1 and drug 2 used in combined treatment to generate the same effect. The combination indexes were classified as synergistic when CI was < 0.9 and antagonistic when it was > 1.1, with CI among 0.9-1.1 indicating additive.

Cellular uptake and intracellular distribution of NCs

For the colocalization assay, $A549^{cisR}$ cells were seeded in glass-bottom dishes at a density of 3×10^4 cells and incubated overnight. The cells were then treated with DiI labeled NC for predetermined time intervals, followed by the incubation with Hoechst

33342 and the Lysotracker DND-26 Green for another 30 min. After wash with PBS, the cells were observed under confocal laser-scanning microscope (CLSM).

To verify that cellular uptake of the NCs was due to the energy-dependent endocytosis, the incubation temperature was decreased to 4°C, and cellular uptake of the NCs was examined. A549^{cisR} cells were seeded in 6-well plates at a density of 2 × 10^5 cells/well and incubated overnight, and then treated with DiI-loaded NCs at 37°C or 4°C for 4 h. For incubation at 4°C, the cells in the 6-well plate were placed into a sealing bag that was prefilled with 5% CO_2, and then the bag with the cells was transferred to a 4°C incubator for a 4-hour incubation. The cells were harvested and rinsed with PBS, and fluorescence intensities were analyzed by flow cytometry.

To investigate the exact endocytotic routine for the NCs, A549^{cisR} cells were pre-incubated with specific endocytosis inhibitors at 37°C, with chlorpromazine at 10 μg/mL, cytochalasin D at 40 μM, and filipin III at 5 μg/mL. Following 60 min of incubation, DiI-labeled NCs were added to the cells and incubated for another 4 h. The cells were harvested and washed with PBS three times. Finally, the cellular uptake was analyzed with flow cytometry.

Cell apoptosis

After being seeded in 6-well plates overnight, cells were treated with Pt$^{(IV)}$-NP, SN38-NP, or the NCs for 24 h. Then cells were harvested by trypsinization and rinsed with PBS. Cells were resuspended with a binding buffer containing Annexin V-FITC/PI double staining. After being incubated at room temperate under the dark condition for 20 minutes, cells were subsequently analyzed by florescence-activated cell sorting (FACS).

Cell cycle distribution

3 × 10^5 A549^{cisR} cells were seeded in 6-well plates and incubated for 24 h to allow attachment. Next, cells were treated with Pt$^{(IV)}$-NP (5 μM), SN38-NP (1 μM), or their combination for another 24 h. Followed by trypsin digestion and twice washes with PBS, cells were then fixed with 75% cold ethanol for 30 minutes at 4 °C. Lately, cells of different groups were stained with propidium iodide (PI) solution at room temperate for 30 minutes. Cell cycle distributions were determined on a flow cytometer, and a total number of 10^4 events were recorded for further analysis.

Western blotting analysis

Whole-cell lysates were prepared by RIPA Lysis Buffer (Beyotime) containing the protease and phosphatase inhibitor cocktails on ice for 20 minutes. The protein concentration of each sample was first quantified by a BCA protein determination reagent and subsequently diluted with sample loading buffer to give a concentration of 2 μg/μL. After boiled on a dry heat block, equal amounts of proteins were electrophoresed on an SDS-10% polyacrylamide gel and transferred onto polyvinylidene fluoride membranes. Membranes were then blocked with 5% skimmed milk in 1 × Tris-buffered saline containing 0.1% Tween 20 for 1 h at room temperature, followed by incubation with primary antibodies at 4 °C overnight and visualization of horseradish peroxidase (HRP) -conjugated secondary antibodies by Bio-Rad ChemiDoc analysis system with enhanced chemiluminescence. Primary and secondary antibodies used for immunoblotting were listed in **Table S1**.

Immunofluorescence Staining Assay

A suspension of 5×10^4 A549^{cisR} cells was seeded in a glass-bottom dish and cultured for 24 h. The cells were treated with Pt$^{(IV)}$-NP, SN38-NP, or the NCs for 24 h. Cells without treatment were used as a control. After a 24 h incubation, the cells were washed three times with PBS and fixed with 4% paraformaldehyde for 30 min at room temperature. The cells were then permeated with 0.5% Triton X-100 in PBS for 15 minutes and blocked with goat serum for 1 h at room temperature. Subsequently, the cells were immunostained with γH2AX antibody (Cell Signaling Technology) at 4 °C overnight. After brief washes, the cells were stained with Alexa Fluor 555-labeled secondary antibody (Thermo Fisher Scientific) for 40 minutes at room temperature, followed by nuclei staining with DAPI for 15 minutes. Finally, the cells were imaged by a Nikon fluorescence microscope (Nikon, Japan).

qPCR analysis

Total RNA was extracted from cell lysates according to the protocol of the manufacturer (YiShan Biotech, Shanghai, China). After that, 1 μg RNA of each sample was converted into cDNA by a fast all-in-one reverse transcription kit (YiShan Biotech, Shanghai, China). The cDNA was then treated as templates for further quantification of mRNA transcription by using a CFX96 Real-Time PCR Detection System (Bio-Rad). All primers were provided by Tsingke Biological Technology (Hangzhou, China) and

their sequences were summarized in **Table S2**. All real-time qPCR analyses were performed in triplicate. Fold change was calculated using the Ct method. The results of the target genes were normalized to β-2 Microglobulin.

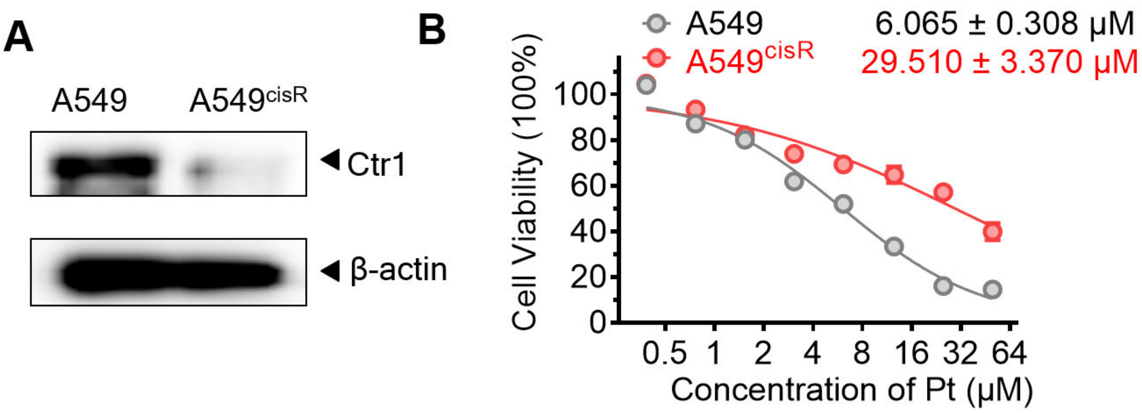

Figure S11. The resistant feature of A549^{cisR} cells was confirmed *via* western blotting and the cell viability assay. (A) Ctr1 expression in A549 and A549^{cisR} cells as determined by western blotting. (B) Cell viability profiles of both sensitive and resistant cells after exposure to varying concentrations of cisplatin.

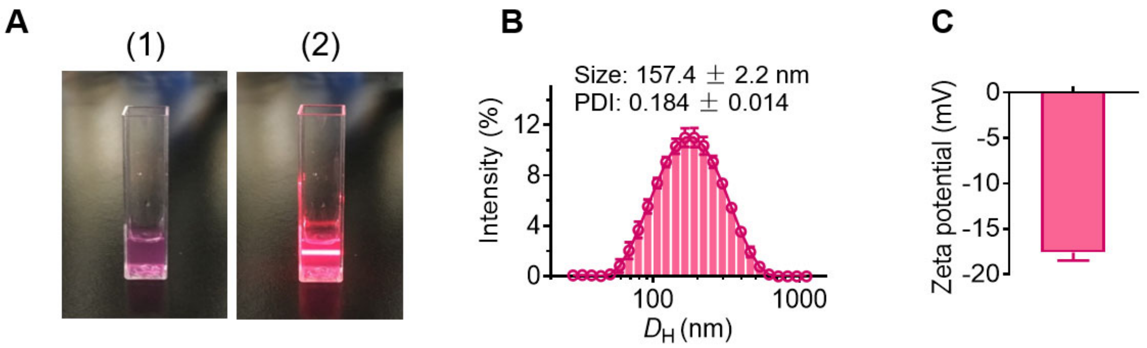

Figure S12. Labeling the NCs *via* the fluorescent dye DiI didn't alter the characteristics. (A) Images showing the appearance of the DiI-loaded NCs in the absence or presence of a laser beam. (B) Size distribution of DiI-labeled NCs as measured by DLS. The DiI-labeled NCs exhibits a hydrodynamic diameter of 157.4 ± 2.2 nm with a low PDI value (0.184 ± 0.014). (C) Zeta potential of DiI-labeled NCs as determined by DLS measurement.

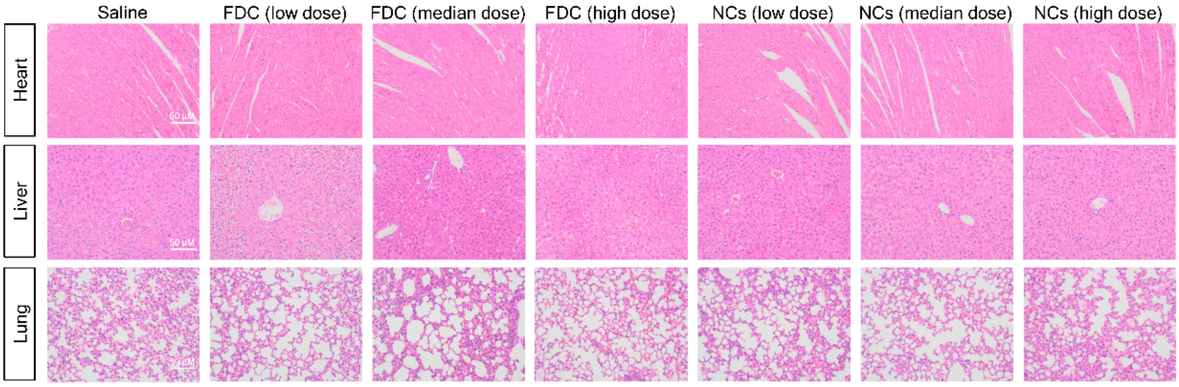

Figure S13. Representative images of hematoxylin and eosin (H&E) staining of heart, liver, and lung after the mice treated with varying dosages of free drug combinations (FDC) and NC regimens.

	Antibody	Dilution	Company Cat # and RRID
Primary antibody	Mouse anti-γH2AX	1:1000 (1:200, IF)	Cell Signaling Technology, Cat# 80312S, RRID: AB_2799949
Primary antibody	Rabbit anti-Rad51	1:10000	Abcam, Cat# ab133534, RRID: AB_2722613
Primary antibody	Rabbit anti-phospho-ATM	1:1000	Cell Signaling Technology, Cat# 13050, RRID: AB_2798100
Primary antibody	Rabbit anti-phospho-Chk2	1:1000	Cell Signaling Technology, Cat# 2197, RRID: AB_2080501
Primary antibody	Mouse anti-phospho-P53	1:1000	Santa Cruz Biotechnology, Cat# sc-56173, RRID: AB_785043
Primary antibody	Mouse anti-P53	1:1000	Santa Cruz Biotechnology, Cat# sc-47698, RRID: AB_628083
Primary antibody	Rabbit anti-cleaved Caspase-3	1:1000	Cell Signaling Technology, Cat# 9664S, RRID: AB_2070042
Primary antibody	Rabbit anti-Caspase-3	1:1000	Cell Signaling Technology, Cat# 14220S, RRID: AB_2798429
Primary antibody	Rabbit anti-PARP	1:1000	Cell Signaling Technology, Cat# 9532S, RRID: AB_659884
Primary antibody	Mouse anti-β-actin	1:2000	Cell Signaling Technology, Cat# 3700S, RRID:

			AB_2242334
Primary antibody	Mouse anti-GAPDH	1:2000	Cell Signaling Technology, Cat# 97166S, RRID: AB_2756824
Secondary antibody	Anti-mouse IgG, HRP linked	1:2000	Cell Signaling Technology, Cat# 7076P2, RRID: AB_330924
Secondary antibody	Anti-rabbit IgG, HRP-linked	1:2000	Cell Signaling Technology, Cat# 7074P2, RRID: AB_2099233
Secondary antibody	Donkey anti-mouse, Alexa Fluor® 555	1:1000	Thermo Fisher Scientific, Cat# A-31570, RRID: AB_2536180

Table S1. Antibodies used for western blotting or immunocytochemistry (IF).

Target	Forward /Reverse primer (5'-3')
53BP1	GTCATTGAGCAGTTACCTCAG/GGGAATGTGTAGTATTGCCTG
BRCA1	AAGGTTGTTGATGTGGAGGAG/CAGAGGTTGAAGATGGTATGTTG
RAD50	CAGACCAGGGACAGACTTGC/CAGCATGGCTCGCTGTTTTG
RPA1	CATTGCGGCCATCATGCAG/GGTTCAACTGTGTCGCCAAC
RAP80	ACATCAAGTCTTCAGAAACAGGAGC/TGCAGCCTGCCTCTTCCAT
XRCC1	CTCCCCCTTTGGCTTGAGTT/GCTGTGACTGGGGATGTCTT
XRCC4	AATCCACCTTGTTTCTGAACCC/CCTTTTTCCATTGCCATGTCATC
β-2-microglobulin	TGCTGTCTCCATGTTTGATGTATCT/TCTCTGCTCCCCACCTCTAAGT

Table S2. The primers were used for the detection of the DNA repair proteins.

Reference:

[1] Fang T, Ye Z, Wu J, Wang H. Reprogramming axial ligands facilitates the self-assembly of a platinum(iv) prodrug: overcoming drug resistance and safer in vivo delivery of cisplatin. Chemical communications. 2018;54:9167-70.

[2] Chou T-C. Drug Combination Studies and Their Synergy Quantification Using the Chou-Talalay Method. Cancer Research. 2010;70:440.
